# What Did We Learn From Current Progress in Heat Stress Tolerance in Plants? Can Microbes Be a Solution?

**DOI:** 10.3389/fpls.2022.794782

**Published:** 2022-05-23

**Authors:** Muhammad Ahmad, Muhammad Imtiaz, Muhammad Shoib Nawaz, Fathia Mubeen, Asma Imran

**Affiliations:** Microbial Ecology Lab, Soil and Environmental Biotechnology Division, National Institute for Biotechnology and Genetic Engineering (NIBGE), Faisalabad, Pakistan

**Keywords:** climate change, heat stress, heat stress effect on plants, heat tolerant PGPRs, sustainable agriculture

## Abstract

Temperature is a significant parameter in agriculture since it controls seed germination and plant growth. Global warming has resulted in an irregular rise in temperature posing a serious threat to the agricultural production around the world. A slight increase in temperature acts as stress and exert an overall negative impact on different developmental stages including plant phenology, development, cellular activities, gene expression, anatomical features, the functional and structural orientation of leaves, twigs, roots, and shoots. These impacts ultimately decrease the biomass, affect reproductive process, decrease flowering and fruiting and significant yield losses. Plants have inherent mechanisms to cope with different stressors including heat which may vary depending upon the type of plant species, duration and degree of the heat stress. Plants initially adapt avoidance and then tolerance strategies to combat heat stress. The tolerance pathway involves ion transporter, osmoprotectants, antioxidants, heat shock protein which help the plants to survive under heat stress. To develop heat-tolerant plants using above-mentioned strategies requires a lot of time, expertise, and resources. On contrary, plant growth-promoting rhizobacteria (PGPRs) is a cost-effective, time-saving, and user-friendly approach to support and enhance agricultural production under a range of environmental conditions including stresses. PGPR produce and regulate various phytohormones, enzymes, and metabolites that help plant to maintain growth under heat stress. They form biofilm, decrease abscisic acid, stimulate root development, enhance heat shock proteins, deamination of ACC enzyme, and nutrient availability especially nitrogen and phosphorous. Despite extensive work done on plant heat stress tolerance in general, very few comprehensive reviews are available on the subject especially the role of microbes for plant heat tolerance. This article reviews the current studies on the retaliation, adaptation, and tolerance to heat stress at the cellular, organellar, and whole plant levels, explains different approaches, and sheds light on how microbes can help to induce heat stress tolerance in plants.

## Introduction

Crop growth is the function of temperature, soil fertility, and water status; all at optimum satisfaction. Temperature is the key determining factor of the vegetation of a particular region (Argosubekti, 2020) and a balance between optimal temperature range and time of incidence is important to regulate plant growth. A slight change/ deviation in the atmospheric temperature, can disrupt normal biological, structural, and molecular processes in plants, which ultimately results in stunted growth and reduced yield (Argosubekti, 2020). Heat stress is an increase in environmental temperature (above the upper limit of threshold) for a certain period that can cause irreversible plant damage. The level of crop adaptation and productivity affected by heat stress, however, depends upon the temperatures and the phase of plant development.

**Figure 1:**
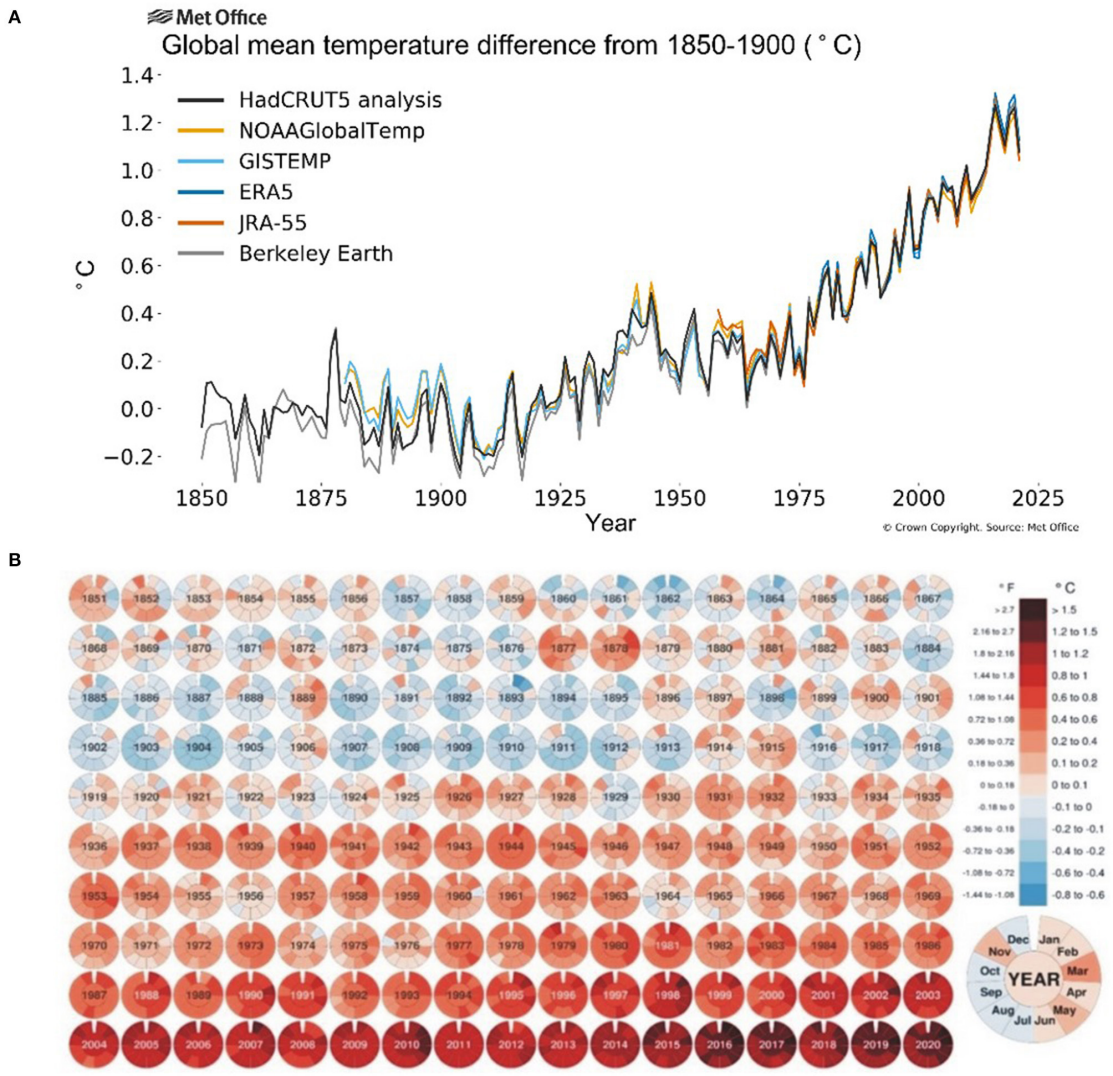
Average global temperatures from 1850 to 2025 compared to a baseline average from 1850 to 1900 **(A)** source: Berkeley Earth (2019); and monthly global mean temperature 1851–2020 compared to 1850–1900 averages **(B)** source: Visual Capitalists (2019).

The onset of industrial revolution and massive mechanization has shown a gradual increase in CO_2_ emissions (30–150%) and greenhouse effect over the past 250 years (Friedlingstein et al., 2010) leading to a persistent rise in temperature during the last two centuries. The global land and ocean surface temperature increased by around 0.85°C from 1880 till 2012 and an annual rise of at least 0.2°C per decade is further anticipated (Change, 2014) while the greenhouse gases will further add 1.1–1.5°C to it. The National Aeronautics and Space Administration (NASA) released a comparative data of average global temperature from with a baseline of 1951–1980 (Figure 1) showing that average monthly temperature has increased >1°C during the past decade (2000–2020) and >1.5°C during the past 5 years (2015–2020). With 1.5–2.0°C change in the global mean temperature, the possibility of suffering from record-breaking, high-impact extreme climate disasters increase significantly, which ultimately cause a significant yield reduction and food supply chain around the globe (Chen et al., 2018; Kong et al., 2018). The IPCC (Intergovernmental Panel on Climate Change) reports that the world's major staple crops and food production have a great impact of climate change (Easterling et al., 2007; Porter et al., 2014) where an increase of one Celsius atmospheric temperature costs a decrease of 6% in crop yield (Asseng et al., 2015). In China, heat stress in the last decade resulted in rice yield losses of 5.18 million tons (Tian et al., 2009) while in South-East Asia upto 14%. In wheat, about 7 million ha in developing countries and 36 million ha in the temperate region had been affected by heat stress in 2001 (Reynolds et al., 2001) causing a yield reduction of 19 million tons (Lobell and Field, 2007) while USDA (United States Department of Agriculture) reported ≈5.5% yield reduction in wheat (Lobell et al., 2011).

Agricultural and global food production is one of the most fragile sectors of society prone to climate uncertainty and changes (Meinke et al., 2006). Detailed multi-locational field analyses have shown that heat and drought stress are more closely linked. Increased heat imposes the drought stress hence, both stresses should be considered together rather than dealing individually. Zia et al. (2021) highlighted the impact of drought along with possible eco-friendly strategies to improve plant growth under drought stress, but the eco-friendly heat-stress management strategies is urgently needed. The plants are substantially affected by high day, low night or high air and surface temperatures. Each cultivar has a temperature optima (threshold) for a significant vegetative and reproductive growth (Zinn et al., 2010; Hatfield and Prueger, 2015) while growth ceases above or below the threshold (Kaushal et al., 2016). The lower threshold temperatures may vary from plant to plant, but is usually lower for most of the tropical, temperate, and cold season crops (Miller et al., 2001). Exposure to high temperature, e.g., 35°C significantly decreases pollen viability (Dupuis and Dumas, 1990), replication of amyloplast and cell division and growth rate ultimately decreasing the size and the total number of the grains (Commuri and Jones, 2001; Rangan et al., 2014). Table 1 describes in detail the growth destructive temperatures of different crops. The higher degree of heat stress (45°C or above) disrupts cellular homeostasis, induces extreme growth retardation, and severely affects the biological activity of proteins due to aggregation or misfolding rendering cells unable to defend themselves (Sarkar et al., 2013; Reddy et al., 2016). The deposition of improper-folded proteins may be permanent that alter the functioning of the cells.

**Table 1:** Growth destructive temperature of different crops.

**Plants**	**Exposure time**	**Destructive temperature (**°**C)**	**References**
**Cereal crops**			
(*Triticum aestivum*) Wheat	10–15 min	45–50	CIMMYT, 2020
(*Zea mays*) Maize, Corn	10	49–51	Argosubekti, 2020
(*Oryza sativa*) Rice	10–15 min	38–45	Sarsu, 2018
**Cash crops**			
(*Gossypium hirsutum)* Cotton	30 min	40–45	Cotton Info, 2018
(*Saccharum officinarum*) Sugarcane	20 min	50–55	Damayanti and Putra, 2010
**Oilseed crops**			
(*Brassica napus*) Rapeseed	10 min	49–50	Argosubekti, 2020
(*Brassica juncea)* Mustard	7 days	40–45	Argosubekti, 2020
**Vegetables**			
(*Solanum tuberosum*) Potato	1 h	42.5	Argosubekti, 2020
(*Allium cepa*) onion	–	30–35	Ikeda et al., 2019
(*Cucurbita pepo*) Squash	10 min	49–51	Argosubekti, 2020
**Fruits**			
(*Citrus aurantium*) Sour orange	15–30 min	50.5	Argosubekti, 2020
(*Vitis vinifera*) Grape	–	65	Argosubekti, 2020
(*Solanum lycopersicum*) Tomato fruits	–	45	Argosubekti, 2020

**Figure 2:**
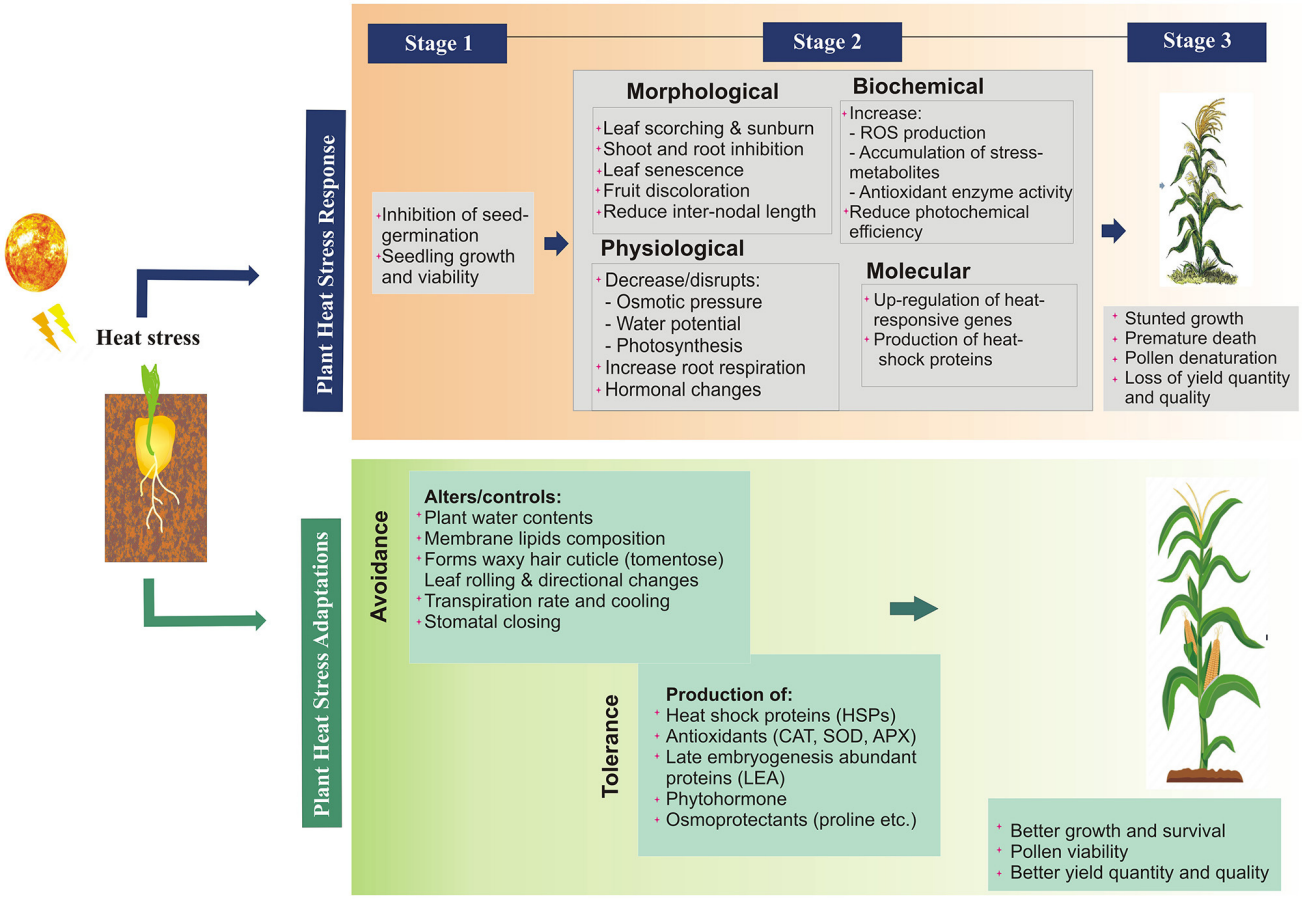
Heat stress responses and adaptation mechanisms in plants at different levels.

The heat stress causes direct and indirect damage to multiple plant functions resulting in morphophysiological changes, hampering different growth phases and metabolic processes (Wu et al., 2018) and ultimately yield reduction (Mcclung and Davis, 2010; Grant et al., 2011). The detail the impact of heat stress on various plant functions at multiple levels has been described in Figure 2. The direct effect includes; protein denaturation and misfolding, increased membrane fluidity, the inactivation of chloroplast and mitochondrial enzymes, inhibition of protein synthesis and degradation, and loss of membrane integrity (Howarth, 2005). Indirect impact includes the changes in the pathogen behavior and disease pattern. Each pathogen has an optimum temperature for its replication and virulence (Velásquez et al., 2018), e.g., *Globodera pallida* nematode infect potato at 15°C (Jones et al., 2017), *papaya ringspot virus* (*PRSV*) infect papaya at 26–31°C (Mangrauthia et al., 2009). It has been predicted that many diseases will migrate into new geographical areas as temperatures rise, where they will encounter new hosts (Etterson and Shaw, 2001), cause severe and frequent epidemics (Ma et al., 2015), improve their survival under heat or desiccation, or become dormant for many years (Turkensteen et al., 2000; Ritchie et al., 2013). Change in the temperature also changes the pathogen behavior (Roberts et al., 2018), e.g., new pathogen strains adapted to high temperatures are being reported which are more active, more virulent, and widely transmitted worldwide (Hovmøller et al., 2008; Milus et al., 2009), e.g., *Phytophthora infestans* (cause late blight in potato and tomato) (Mariette et al., 2016) and *Puccinia striiformis* (rust fungus in wheat). Even pathogens can cause an outbreak by transient variations in temperature, e.g., soybean rust can be developed even after 1-h of exposure at 37°C, although, the optimum temperature for disease development is 12–25°C (Bonde et al., 2012).

Plant endophytic bacteria have a symbiotic relationship with their host plant, dwelling within it for the bulk of their life cycle and having the capacity to colonize the plant's interior tissues *via* penetrating the seed and root. These endophytic microbiomes fixed the atmospheric nitrogen, produced phytohormones, solubilized the inorganic phosphorus, zinc, potassium and calcium, produced exopolysaccharides and iron chelating siderophore compounds under heat stress condition which ultimately enhance the plant growth (Hakim et al., 2021). On the other hand, there are also some beneficial fungi, i.e., arbuscular mycorrhiza fungi (AMF) that is also help plants under heat stress condition. These AMF form symbiotic relationship with plant roots and improve photosynthetic process, increase nutrients uptake, secondary metabolites accumulation, regulate the oxidative defense system, and maintain the osmotic balance in plants (Begum et al., 2019). Plant genetic engineering and maker assisted selection breeding and multiple approaches have been published multiple reviews on heat stress and their management which are costly and time consuming. On the other hand, PGPR is cost-effective, eco-friendly, and ecologically safe solution to induce heat stress tolerance. So, current review will give a quick rundown of the current knowledge about the methods and pathways that PGPR use to promote tolerance in plants under heat stress. It will also discuss the heat stress response factors and genes and how PGPR regulate the expression of such genes to modify plant response under heat stress.

## Plant Response to High Temperature

The response of plants toward heat stress varies with the degree and duration and the plant's developmental stage (Ruelland and Zachowski, 2010). These heat stress disorders may be recurrent or persistent in nature or both, therefore, plants have evolved different mechanisms to cope with them (Bäurle, 2016). The heat stress response (HSR) is an evolutionarily conserved mechanism that describes the plant's adaptation and induction of thermotolerance by either by activating the defense mechanisms to stop the disruption at the transcriptomic level in the cell or by the activation of heat shock proteins (HSPs), epigenetic pathways, and DNA methylation (Lämke and Bäurle, 2017). Table 2 describes few major effects of heat stress on plants.

**Table 2:** Effect of heat stress on different stages of plant growth in major cereals.

**Plants**	**Developing stage**	**Temperature**	**Major effect**	**References**
(*Triticum aestivum*) Wheat	60 days after sowing	30/25°C day/night	Reduce leaf size, elongation of booting stage, heading stage, pollination process.	Djanaguiraman et al., 2010
	Maturity and Grain filling	37/28°C (day/night), 20 days		Rahman et al., 2009
			Decrease the number of spikelets and reduce final yield. Early maturation and grain filling.	
			Decreased grain weight.	
	Vegetative stage	25–42.5°C	Reduce CO_2_ concentration within plants.	Djanaguiraman et al., 2011
(*Oryza sativa*) Rice	Panicle stage	Above 33°C, 10 days	Reduced the rates of pollen and spikelet fertility.	Hurkman et al., 2009
	Reproductive stage	32°C (night temperature)	Enhance pollen sterility, decrease grain size and weight which leading to yield reduction.	Suwa et al., 2010
(*Zea mays*) Maize, Corn	Reproductive stage	35/27°C (day/night), 14 days	Suppress the production of cellulose and hemicellulose which leading to a reduction in photosynthate supply in plants	Yin et al., 2010
	During pre-anthesis and silking onwards	33–40°C, 15 days	Decrease plant growth and ear formation rate.	Zhang et al., 2013

### Morphological Response During Early and Reproductive Growth

Of all the growth phases, seed germination is the most adversely affected phase by heat stress, but, the response varies in different crops or even within varieties (Sita et al., 2017). The germination is significantly reduced above 45°C because of denaturation and embryonic cells (Cheng et al., 2009), along with severe impact on seedlings potency, the development of radicle and plumule, and seedling growth (Toh et al., 2008; Sita et al., 2017). At later stage, the effect varies with the time, length, and severity of stress (Fahad et al., 2016) but mainly heat stress decreases the cell water contents, the cell size, the plant size, growth and biomass, net assimilation rate (NAR) and relative growth rate (RGR) (Ashraf and Hafeez, 2004; Wahid and Close, 2007). Other visual symptoms include leaves and twigs scorching, growth inhibition, damaged leaves, early leaf senescence, discoloration of fruits/plants (Vollenweider and Günthardt-Goerg, 2005), reduced number of floret and spikes (Prasad et al., 2006; Fahad et al., 2016), decrease in the inter-nodal length (Siddiqui et al., 2015; Gray and Brady, 2016), plant height, total biomass, and the number of panicles (Modarresi et al., 2010). At the reproductive stage, one degree rise in temperature is detrimental because it degenerates mitochondria and proteins, loss of seed mass, quality, viability and vigor (Nahar, 2013; Balla et al., 2019).

### Physiological Responses

#### Water and Nutrient Uptake

The heat usually correlates with water because it cause dehydration in plant tissues especially in tropical and subtropical ecosystems (Giri, 2013; Giri et al., 2017). High temperature is lethal under both insufficient (Giri et al., 2017) or sufficient soil water because it affect the water conductivity and root permeability (Giri, 2013) which ultimately restrict the water and nutrients supply to the plant causing starvation and reduced growth (Wahid et al., 2007; Huang et al., 2016). The exact basis of the crop nutrient relationships under heat is not clearly known but overall, it lowers the photosynthesis and water viscosity and the activity of enzymes, e.g., nitrate reductase that is involved in the breakdown of nutrients, recycling, absorption, and accessibility to the plants (Kumarathunge et al., 2019).

#### Photosynthesis

Photosynthesis is among the most thermosensitive pathways in plants which is adversely affected due to the heat-induced reduced water contents, numbers of leaves, premature senescence and cell death (Hu et al., 2020). The major impact is the heat-induced injury to the photosynthetic machinery, i.e., stroma and thylakoid lamellae (Kmiecik et al., 2016; Sun and Guo, 2016). This disruption inhibits the thylakoid activity and the functional efficiency of photosystem II (Morales et al., 2003). Other plant processes that hamper photosynthesis under thermal stress include the reduction of photosynthetic pigments (Marchand et al., 2005), leaf moisture, transpiration rate, low CO_2_ concentration and supply to photosystem due to stomatal closure (Ashraf and Hafeez, 2004), reduction of amino acids, rubisco binding proteins (Li et al., 2018), carbohydrate depletion and plant malnutrition due to reduced activity of ADP-glucose pyro-phosphorylase, sucrose phosphate synthase, and invertase (Djanaguiraman et al., 2009). The reduction in chlorophyll pigment is linked with the activity of thylakoid membranes, lipid peroxidation of chloroplast, and reduced photochemistry (*F*_v_/*F*_m_ ratio) of photosystem II, which ultimately reduce the overall photosynthesis in crop plants (Mohammed and Tarpley, 2010). Reduction of chlorophyll may be due to the reduced (about 60–90%) biosynthesis under high or low temperature, or increased chlorophyll-pigments degradation, or an accumulative effect of the two (Hemantaranjan et al., 2014).

#### Oxidative Damage

Heat exposure stimulates oxidative stress due to the generation of activated, extremely reactive, and toxic oxygen species. The reaction of reactive oxygen species (ROS) includes supper oxide radical (O${}_{2}^{-}$) singlet oxygen (O_2_), hydroxide ions (hydroxyl) radical (OH^−^), and hydrogen peroxide (H_2_O_2_) (Marutani et al., 2012; Suzuki et al., 2012). ROS causes lipid membrane to lose the ability to regulate substance exchange across the cell membrane (Suzuki et al., 2012). Oxidative stress enhance peroxidation and further damage the proteins, lipids, carbohydrates, and DNA contributing to premature aging (Savicka and Škute, 2010). Continuous thermal stress increases the ROS deposition in the plasma membranes with cell membrane depolarization, which leads to the activation of the RBOHD enzyme (respiratory burst oxidase homolog protein-D) that produces ROS and initiates programmed cell death signal (Mittler et al., 2011). The elevated temperature raises the O_2_ content in the root by 68%, and leaf malondialdehyde (MDA) content by 27% at the early and 58% at later stages (Medina et al., 2021). Though, the plant has a special mechanism to avoid unnecessary reactive oxygen species (ROS) by the production of specific antioxidants.

#### Respiration

Heat stress affects mitochondrial functions by influencing respiration. The respiration often rises with increasing temperature, but at a certain duration of photoperiod, the respiration process declines due to damage to the respiratory system (Prasad et al., 2008; Rasmusson et al., 2020). In the heat-prone varieties under heat stress (35/25°C day/night) the respiration rate in the flag leaf of wheat was considerably higher compared to that of the control (23/18°C day/night) (Akter and Islam, 2017). The solubility of O_2_ and CO_2_ as well as Rubisco's kinetics, loss of respiratory carbon due to heat enhances the ROS and reduces ATP production and respiration in plants (Cossani and Reynolds, 2012).

### Crop Yield Response

HS induces significant yield reduction mainly due to decrease in the number, size and quality of grain, starch synthesis and accumulation, protein concentration, pod, fiber content, and breakdown of the Ca content (van Es, 2020). HS usually accelerates the rate of grain-filling but shortens the grain-filling time leading to significant decrease in grain length, width, and weight, grain quality (Högy et al., 2013; Lamaoui et al., 2018) reduced accumulation of storage compounds (Hurkman et al., 2013) and increased male sterility (Suwa et al., 2010). HS also increase proteinogenic amino acid and maltose content and decrease the concentrations of starch, sugars, raffinose, sucrose and lipids (Vasseur et al., 2011). A slight increase in temperature (1–1.5°C) reduces the harvest index and yield up to 10% in different crops (Table 3) (Tubiello et al., 2007; Ahamed et al., 2010; Hatfield et al., 2011). Heat-susceptible cultivars show more yield losses compared with thermo-tolerant cultivars (Ahamed et al., 2010; Hussain et al., 2019).

**Table 3:** Yield losses reported in different crops due to heat stress.

**Plants**	**Yield loss (%age)**	**References**
**Cereal crops**		
(*Triticum aestivum*) Wheat	18–30	Balla et al., 2011; Djanaguiraman et al., 2020; Dubey et al., 2020
(*Zea mays*) Maize, Corn	42	
(*Oryza sativa*) Rice	50	Li et al., 2010; Da Costa et al., 2021; Xu et al., 2021
**Cash crops**		
(*Gossypium hirsutum*) Cotton	50	Zafar et al., 2018; Majeed et al., 2021
(*Saccharum officinarum*) Sugarcane	20–40	Zhao and Li, 2015; Hussain et al., 2018
**Vegetables**		
(*Solanum tuberosum*) Potato	12–35	Rykaczewska, 2015; Momčilović, 2019
(*Allium cepa*) onion	20–50	Kandil et al., 2011; Ratnarajah and Gnanachelvam, 2021

## Plant Heat-Adaptation Strategies

Plant HS adaptation involves a variety of strategies and various processes including basal heat tolerance (BHT), acquired heat tolerance (AHT), and avoidance (Fitter and Hay, 2012; Bäurle, 2016). *BHT* is the natural capacity of plants to tolerate heat while *AHT* (also called priming or acclimation), is acquired tolerance *via* short pre-exposure to heat (Yeh et al., 2012). A sudden heat exposure results in short-term reaction, i.e., leaves orientation, osmotic modification, evaporation, and adjustments in cell membrane structure (Bäurle, 2016; Zhongming and Wei, 2021) whereas, under long-term exposure, multiple adaptation mechanisms work synergistically to minimize the impact of heat stress. Heat tolerance involves the activation of some important immunity pathways, including ions transporters, proteins late embryogenesis abundant (LEA), phytohormones, antioxidant-resistance, and factors implicated in transduction signaling and transcriptional control (Li, 2020; Li et al., 2021). AHT is distinguished between short-term acquired tolerance (SAT) and long-term acquired tolerance (LAT) and establish a molecular stress memory state that protect themselves from acute heat damage and death (Sani et al., 2013). The stress-sensitive feature is characterized by an early signal that can be in the form of anionic and osmotic effects or alterations in membrane fluidity which restore homeostasis and sustain defective proteins and membranes (Kumarathunge et al., 2019). Plants HS *avoidance* includes long-lasting developmental morphological and physiological adaptations or short-lived accommodation strategies (Figure 2), e.g., intensive transpiration from leaves prevents damage by lowering the temperature of the leaves by 6°C or even 10–15°C than the normal temperature (Fitter and Hay, 2012; Li, 2020). Other plant varieties have special features that enable them to escape from warm conditions, e.g., having heat-sensitive buds, leaf abscission, annual desert buds, and by completing the entire regenerative period during the winter (Fitter and Hay, 2012). These adaptations are correlated to each other and increases the net process of photosynthesis at HS (especially for C4 and CAM plants) (Sarieva et al., 2010; Li, 2020).

### Antioxidant System

Plants have evolved a series of detoxification systems to fight against oxidative damage. These systems include peroxidase (POX), ascorbate peroxidase (APX), glutathione reductase (GR), superoxide dismutase (SOD), and catalase (CAT) which show mitigating effects against different kinds of stress including HS (Suzuki et al., 2012; Caverzan et al., 2016). These antioxidants prevent the excessive free radicals and repair the damage effects that function as a catalyst to change the dismutation reaction from SOD anion to H_2_O_2_ and O_2_ molecules. They also affect other physiological phenomena including biosynthesis of lignin, enzyme catabolism, defense against injury and insect/pathogen attack, and physiological damage caused by temperature stress (Wani et al., 2016; Devireddy et al., 2021).

### Production of Metabolites and Hormones

The production of a wide range of metabolites of low molecular mass including soluble carbohydrates, amino acids, variety of sugars, and sugar alcohols and phenolics have been linked to stabilization of cellular membranes, protection of protein structures, maintenance of the cell water balance, and buffering of the cellular redox potential under abiotic stress in plants (Kumarathunge et al., 2019). Accumulation of different osmoprotectants (e.g., proline, glycine betaine, etc.) and carotenoids (e.g., xanthophlls, terpenoids, etc.) under extreme temperature participate directly in the osmotic adjustment and show positive correlation with more negative leaf osmotic potential and production of protective pigments (Wani et al., 2016; Li et al., 2021). Glycine betaine maintains the activity of Rubisco by preventing its thermal inactivation (Devireddy et al., 2021). Sucrose and other carbohydrates act as antioxidant (Lang-Mladek et al., 2010; Devireddy et al., 2021) as well as regulate carbon allocation and sugar signaling consequently protecting the pollen viability. Plant growth regulators are also involved in enhancing plants' ability to tolerate stress, e.g., ABA, SA, IAA or CK (Ding et al., 2010; Hsu et al., 2010; Devireddy et al., 2021).

### Heat Shock Factors, Proteins and CRISPR Technology

**Figure 3:**
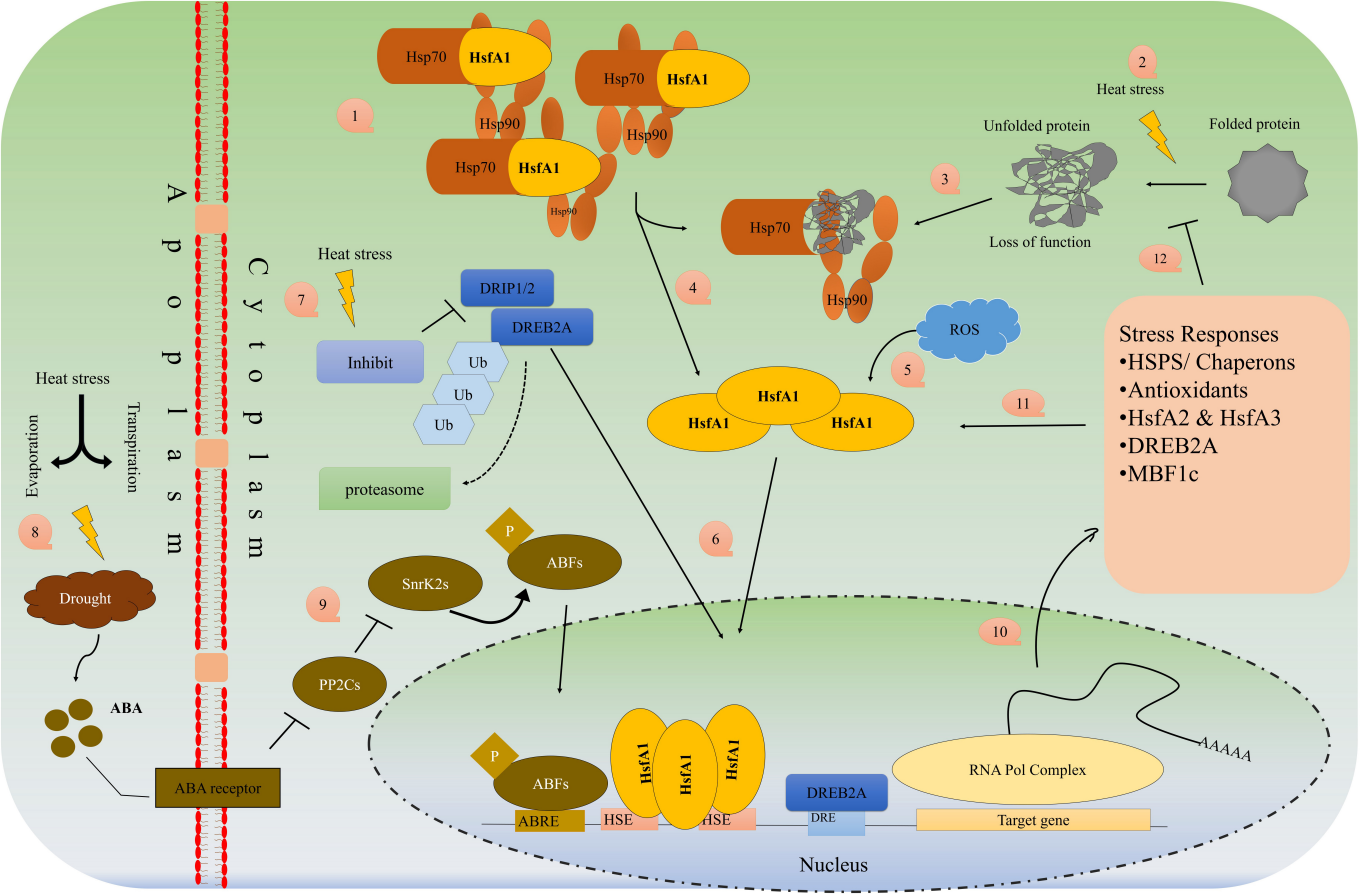
Schematic overview of HSP/HSF's pathway activation under heat/drought stress.

Many stress-inducible transcriptions factors (known as heat shock factors; HSF, e.g., Hsf6A, DREB1A, OsMYB55), stress-related genes, and proteins (HSP) are synthesized and overexpressed to induce thermotolerance (Lamaoui et al., 2018). The HS response is preceded by HSF that acts further on the transcription of HSP mRNA. HSFs are components of a complex signaling system that control responses not only to high temperatures but many other abiotic stresses. They are transcriptional activators of HS genes and bind specifically to heat shock sequence elements throughout the genome. Figure 3 describes in detail the mechanism of HSP/HSF activation under heat stress in the plant cell. A rapid increase in temperature up to 5–10°C, causes the plant to trigger the “heat-responsive genes” which translate into a special protein called heat shock proteins (HSPs). HSPs protect the plants by activating the chaperons and proper folding of proteins, inhibit the denaturation and aggregation of intercellular proteins and maintain their function by proper folding of proteins (Baniwal et al., 2005). HSPs are miscellaneous, highly diverse and normally present in living cells but under heat stress, different small and large HSPs are produced which help in (i) folding of newly synthesized proteins (Hsp60 and Hsp70), (ii) translocation the proteins between the cellular membrane and from organelle to organelle (Hsp70), (iii) prevent from deterioration, self-aggregation, improper folding, and the production of the polymeric compound of proteins (sHsp, Hsp70, Hsp90, Hsp100), (iv) the breakdown of defective proteins by proteolytic (Hsp70, Hsp100), (v) activation of signaling molecules, transduction, transcriptional factors and transcription (Hsp70, Hsp90), (vi) production of oligomeric complexes of high molecular weight that act as a cellular matrix for unfolded protein stabilization (Hsp20 or small Hsp (sHSP) (Hasanuzzaman et al., 2013).

CRISPR/Cas9 is a genome editing tool, widely use to edit the eukaryotic genome specially in plants The transient expression of this system is very useful to activate or limit the desired targeted gene in plants (LeBlanc et al., 2018). Recently, the scientist explained the heat-shock inducible CRISPR/Cas9 system for genome editing and demonstrate its effectiveness in rice. They combined the promoter of heat shock protein of rice *U3* and soybean to induce the HS-CRISPR/Cas9 system *via* Cas9 and single guider RNA (Nandy et al., 2019). In another study, the Enhanced response to ABA1 (ERA1) gene present in *Arabidopsis thaliana* was targeted, that encode the farnesyltransferase and upregulate the abscisic acid (ABA) signaling under heat and drought stress. By using CRISPR/Cas9 system, they knock in the *ERA1* gene into rice genome and enhanced the ABA in rice under heat and water deficit condition (Ogata et al., 2020).

## Managing Agriculture Under Heat Stress

By proper agronomic management, such as water, the methods and quantity for fertilization, the sowing time and method, the addition of protectants, plants can be grown effectively under heat stress (Ortiz et al., 2008). The usual practices are as follows:

- *Mulching* is done to preserve optimum moisture in soil, because it reduces soil evaporation (Chen et al., 2015), improves nitrogen and water efficiency (Singh et al., 2011), prevents yield loss (Chakraborty et al., 2008; Głab and Kulig, 2008) in temperate and tropical regions under thermal stress (Akter and Islam, 2017).
- Proper *sowing time* is very important for crops as early or late plantation shortens the heading and maturity period, improves pathogen infestation, eventually affecting economic yield and grain quality (Khichar and Niwas, 2007; Al-Karaki, 2012; Hakim et al., 2012; Hossain and Da Silva, 2012).
- Increasing *crop nutrition* (e.g., N, Ca, K, Mg, B, Se, and Mn) alter the stomatal function, activate physiological and molecular processes, improve tissue water potential, decrease ROS toxicity by increasing the antioxidant concentration stimulate heat stress tolerance (Waraich et al., 2012; Mengutay et al., 2013). The post-anthesis foliar application also improve grain proteins, slows down the ABA synthesis, improves cytokinin production, stimulates the photosynthesis and accumulation of assimilates (Dias and Lidon, 2010; Singh et al., 2011).
- The exogenous application of *growth promoters* [e.g., phytohormones, osmoregulatory, receptor molecules, polyamines along with spermidine, putrescine, spermine, putrescine, alpha-tocopherol (vitamin E), arginine etc.] regulate the ROS and enhance the efficiency of the antioxidant thus protect plants under HS (Farooq et al., 2011; Sharma and Chahal, 2012; Hemantaranjan et al., 2014; Uprety and Reddy, 2016).
- Exogenous *calcium application* activates MDA that enhances the activity of guaiacol peroxidase, CAT, SOD, which ultimately induce heat resistance in plants. It can also protect chlorophyll from solar radiation destruction and sustain stomatal functioning (Dias and Lidon, 2009; Waraich et al., 2012). Activation of different enzymatic, e.g., CAT, SOD, ascorbate peroxidase (APX), and non-enzymatic antioxidants, e.g., tocopherol, and ascorbic acid have a significant effect on oxidative management under multiple stresses (Balla et al., 2007).
- Plant treatment with reactive short-chain leaf volatiles (*RSLVs* also known as oxylipins; a group of C4–C9 straight chain carbonyls categorized by an alpha, β-unsaturated carbonyl bond) show high thermotolerance (Yamauchi, 2018).
- Co-polymers of Poly (N-isopropyl acrylamide) (Dimitrov et al., 2007) or poly acrylic acid (PAA) have been used as thermo-tolerant nanoparticle to transport specific chemicals within the plant to develop resistant against HS (Xu et al., 2015).
- The use of natural *bio-stimulants*, e.g., amino acids, microorganisms, fruit extracts, seaweeds, inorganic materials, and chitin or engineered, e.g., plant hormones, phenols, salts, chemical substances, and other elements with plant regulating properties (Calvo et al., 2014; Van Oosten et al., 2017).
- Transfer of heat shock regulatory proteins (HSFs, HSPs) or development of heat shock transcriptional elements (HSEs) enhance gene expression within minute and induced heat tolerance in plants.

## Microbe Therapy for Heat Tolerance

Plants are home to large collection of microbes collectively known as plant microbiome (Liu and Tan, 2017; Álvarez-Pérez et al., 2019) which is highly dynamic in nature and show significant shift in the composition in response to external stimuli and environmental stresses, e.g., heat (Santos-Medellín et al., 2017; Timm et al., 2018). This shift is not a passive plant reaction, but a deliberate response because both plants and microbes have been coevolved since millions of years (Durán et al., 2018; Kwak et al., 2018). Plants use precise combinations of chemical stimuli under abiotic, or pathogen-induced stress which trigger specific microbes to overexpress particular proteins or enzymes for inducing stress tolerance (Bakker et al., 2018; Kumar and Verma, 2018; Liu and Brettell, 2019; Hakim et al., 2021). Overall, the plant microbiome controls nutrient availability, root growth, plant yield and modulates resistance to stresses (Chialva et al., 2018; Lu et al., 2018).

The microbial application (inoculation) is cheap, eco-friendly, low-input, and time-saving strategy as compared to the development of stress-tolerant crop variety or germplasm screening (Shrivastava and Kumar, 2015). Plant growth-promoting microbes (PGPM) require optimum condition for maximum efficiency (e.g., production of phytohormones, nitrogen fixation, and solubilization of nutrients P, Zn, Ca, Fe, etc.). Even though stress factors (salinity, drought, heat, and heavy metals) decrease their efficacy, but some PGPR modify themselves for maximum efficiency under stress. Species of *Enterobacter, Acetobacter*, and *Pseudomonas* solubilize the phosphorus 74%, 75%, and 80% respectively at normal (30–32 ± 2°C) and high temperature (38–40 ± 2°C) (Kachhap et al., 2015). Similarly, the *Acetobacter* spp. produced 100%, *Enterobacter* spp. produced 82% and *Pseudomonas* spp. produced 50% IAA both at high and low temperatures. While high temperature reduced the efficacy of nitrogen fixation in rhizobia. High temperature reduced the symbiotic relationship between plant roots and microbes which ultimately decreased the rate of nitrogen fixation. Heat stress inhibits microbial adherence on root hair and root nodules (Alexandre and Oliveira, 2013) and also disturb the molecular signals between microbes and root hair which leads to forming a weak symbiotic relationship between partners (Sadowsky, 2005). However, some rhizobial species from *Acacia* have thermotolerance up to 44°C which enable them to fix nitrogen at high temperature and give benefit to plant (Zahran et al., 1994).

PGPM are stable under chilling and heat stress (Barka et al., 2006; Ali et al., 2011) but the molecular and physiological changes connected to this stress management are poorly understood. Inoculation studies reports that systemic effects are involved during heat/chilling stress that change metabolic and regulatory function of plant supporting both growth and stress management (Abd El-Daim et al., 2019). Table 4 describes the role of PGPR in plant under stress condition. Decrease in the ROS production by seed treatment with *Bacillus amyloliquefaciens* and *Azospirillum brasilense* has also been reported under HS (Abd El-Daim et al., 2014). A recent review published the meta-analysis of microbe-mediated thermotolerance in plants and their mechanisms from 39 published research articles (Dastogeer et al., 2022). They have reported a significant decrease in accumulation of MDA and H_2_O_2_ in colonized plants showing lower oxidation activity but a corresponding increase in the activities of catalase, peroxidase, glutathione reductase under heat stress. However, the activities of SOD, ascorbate oxidase, ascorbate peroxidase and proline were variable. The overall impact of microbial colonization was more pronounced under heat stress.

**Table 4:** Role of PGPR in plant under stress condition.

**Bacterial strain**	**Crop**	**Role in plant**	**References**
**1-ACC Deaminase**
*Klebsiella* sp.	*Triticum aestivum* (Wheat)	*Klebsiella* sp. SBP-8 protects the plants against adverse effects of salt and heat stress; reduce stress-induced ethylene and regulation of ion transporters	Singh et al., 2015
*Bacillus cereus*	*Triticum aestivum* (Wheat)	Increase plant growth (root, shoot fresh and dry weight, chlorophyll contents) under heat stress	Ali et al., 2011
*Pseudomonas putida*			
**2-Exopolysaccharides**
*Bacillus cereus*	*Solanum lycopersicum* (Tomato)	Increase the number of flowers and fruits Increase chlorophyll, proline, and antioxidants	Mukhtar T. et al., 2020
*Bacillus amyloliquefaciens* UCMB5113	*Triticum aestivum* (Wheat)	Increase HSP26 and chlorophyll content	Abd El-Daim et al., 2018
		Accumulate GABA and modulate metabolic pathways	
*Azospirillum brasilense* NO40			
*Pseudomonas* sp. AKM-P6	*Sorghum bicolor* (Sorghum)	Enhance tolerance of sorghum seedlings to elevated temperatures	Ali et al., 2011
*Rhizobium* sp. (Cajanus)	*Leguminosae* (Legume)	Heat shock protein (Hsp) of 63-74 kDa	Sutherland, 2001
*Pseudomonas* sp. PsJN	*Solanum tuberosum* (Potato)	Promote growth	Bensalim et al., 1998
*Bacillus aryabhattai* H26-2 and *Bacillus siamensis* H30-3	*Brassica oleracea var. capitata* (Cabbage)	Leaf abscisic acid (ABA) content and reduced stomatal opening after stresses treatments, Biocontrol activity against soft rot	Abd El-Daim et al., 2018
*Bradyrhizobium diazoefficiens* USDA110	*Glycine max* (Soybean)	Survival in starvation	Nishihata et al., 2018
*Shinorizobium meliloti*	*Medicago sativa* (Alfalfa)	Affect symbiosis during heat stress	Ogden et al., 2019

### Mechanistic Interpretation of Microbe Therapy

**Figure 4:**
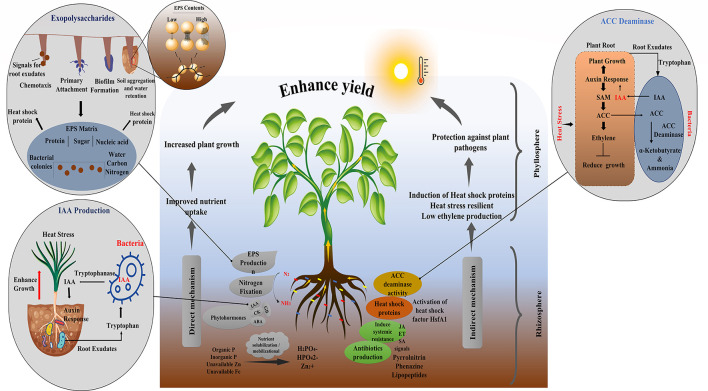
Mechanism of PGPR for growth promotion and a(biotic) stress tolerance.

A variety of PGPM have been known to induce heat stress using various mechanisms (Dodd and Pérez-Alfocea, 2012; Ramadoss et al., 2013). For instance, *Pseudomonas putida* produces heat shock protein that plays a vital role in increasing plant thermal tolerance (Ali et al., 2011) (Figure 4). Many of PGPM activate structural changes in plants that impart tolerance to heat stress, a phenomenon known as induced systemic tolerance (Yang et al., 2009). Apart from inducing direct stress tolerance, several plant-beneficial traits exhibited by these bacteria support plant growth in a synergistic manner under stress (Etesami and Beattie, 2017; Imran et al., 2021). They benefit plants either directly, through the phytohormones production, nutrient mobilization, and nitrogen fixation or indirectly by triggering the signaling cascades in the host plant. *Burkholderia phytofirmans* strain *PsJN* is a well-reported PGPR that enhances heat tolerance in tomatoes, cold tolerance in grapevine, water stress tolerance in wheat, salt, and freezing tolerance in *Arabidopsis*. The same bacterium also has the antifungal property that protects the plant from biotic stress (Issa et al., 2018) revealing that a single bacterium can induce multiple benefits in same or different hosts (Imran et al., 2021).

#### Role of Microbial Phytohormones Under HS

Phytohormones are the key regulators of plant development, and the plants have a natural ability to synthesize, perceive and respond to these hormones. This response is modulated upon exposure to external/internal stimuli (Khan et al., 2014; Hakim et al., 2021), e.g., decreased levels of auxin, cytokinin, and gibberellin and increased ABA level under HS (Wu et al., 2016). One of the mechanisms of PGPM-mediated plant growth improvement is the phytohormone production trait (Etesami et al., 2015) which affect the metabolism of endogenous (Hashem et al., 2016; Sorty et al., 2016) and ultimately play a key role in modulating the plant's response under stress, uptake etc. (Spaepen et al., 2008; Khan et al., 2011). These facts validate that the phytohormones-producing PGPM reverse the adverse effects of heat stress.

Auxin (IAA) is the most important hormone, imperative for cell division, differentiation that controls seed germination, roots development, and apical dominance (Maheshwari et al., 2015). The majority of the rhizospheric microbes (>80%) synthesize and release IAA in the rhizosphere which elongates plant roots, increases the number of root hairs for enhanced uptake of water and nutrients under normal (Oleńska et al., 2020) as well as stress (Imran et al., 2015; Nawaz et al., 2020; Zia et al., 2021). IAA producing *Ochrobactrum* sp. and *Pseudomonas* sp. (volcanos isolates) have shown improved root and shoot length, fresh weight, and biomass of maize under high temperature (40°C), drought (up to 60% Poly Ethylene Glycol 6000), and salt (500 mM NaCl) (Mishra et al., 2017).

Gibberellins (GAs) regulate plant developmental processes such as embryogenesis, leaf expansion, stem elongation, flowering, and fruit ripening (Binenbaum et al., 2018) while abscisic acid (ABA) regulates cell division and elongation, seed dormancy and germination, embryo maturation, floral induction, and responses to stresses (Finkelstein, 2013). PGPM capable to synthesize gibberellins stimulate plant growth and stress tolerance by modulating the endogenous levels of GAs and ABA. For instance, inoculation with GAs producing *Serratia nematodiphila* and *B. tequilensis* increase the endogenous synthesis of GA4 and ABA while reduced the salicylic and jasmonic acids and improved plant biomass under HS (Kang et al., 2015). Phytohormones-producing endophytic fungi (*Phoma* and *Penicillium* sp.) and *Bacillus* spp. also modulate the level of endogenous abscisic acid, salicylic acid, and jasmonic acid under multiple stresses and improve thermotolerance (Waqas et al., 2012). Inoculation with a multiple hormone (IAA, CK, JA, SA, GAs, and ABA) producing *B. aryabhattai* strain significantly improved nodule number, overall plant growth, and increased stress tolerance of soybean to drought and high temperatures (38°C) (Park et al., 2017).

Cytokinins are involved in processes such as seed germination, apical dominance, roots development, nodule organogenesis, development of vascular tissues, flower and fruit, and plant-pathogen interactions (Osugi and Sakakibara, 2015). Different microbes (such as *Bacillus, Escherichia, Agrobacterium, Methylobacterium, Proteus, Pseudomonas*, and *Klebsiella*) inhabiting plant rhizosphere are capable to synthesize and release CK in the rhizosphere which stimulate plant growth under stress including heat (Liu et al., 2013). Exogenous application of INCYDE-F (an inhibitor of CK-oxidase/dehydrogenase) in *Arabidopsis* increased the contents of CK trans-zeatin and cis-zeatin in roots and IAA in all tissues after HS. It further reduced the level of ABA in leaves and ethylene in apices of roots which shows that inhibition of CK-degradation helped the *Arabidopsis* to cope with HS (Prerostova et al., 2020).

Ethylene is a gaseous hormone involved in abscission, senescence, reproductive development, and a(biotic) stress response (Liu et al., 2021). Pollen development is the most thermosensitive stage during reproduction, therefore, regulation of ethylene signaling in reproductive tissues is critical to gain reproductive success (Jegadeesan et al., 2018). Various abiotic and biotic stressors enhanced the levels of ethylene in plant tissues which is detrimental for plants. *Enterobacter* sp. SA187-induced thermo-tolerance to wheat in field has been reported (Shekhawat et al., 2021) which is mediated by the ethylene signaling *via* the TF EN13 and constitutive H3K4me3 modification of HS memory genes, generating robust thermotolerance in plants.

Biosynthesis of ethylene is undertaken by two transcripts (i) *PsACS* [encode enzymes that convert S-adenosyl-L-methionine to 1-aminocyclopropane-1-carboxylic acid (ACC)] and (ii) PsACO (encode enzymes that convert ACC to ethylene (Savada et al., 2017). Various microbes, i.e., *Methylobacterium, Bacillus, Alcaligenes, Enterobacter, Pseudomonas, Azospirillum, Rhizobium*, and *Bradyrhizobium* have an enzyme “1-aminocyclopropane-1-carboxylate (ACC) deaminase,” which metabolize ACC (an immediate precursor of ethylene) into α-ketobutyrate and ammonia ultimately lowering down the ethylene levels and detrimental impact on plants under stress including HS (Saleem et al., 2007). *Burkholderia phytofirmans, Pseudomonas frederiksbergensis, P. vancouverensis, P. putida*, and *B. cereus* are few examples of heat resilient microbes that utilize the ACC-deaminase enzyme to reduce the endogenous ethylene levels in plants produced under a range of different stresses including salinity (Liu et al., 2021), drought (Danish et al., 2020), and heat (Mukhtar S. et al., 2020).

#### Role of Microbial Exopolysaccharides Under HS

Exopolysaccharides (EPSs) are bacterial extracellular polymers that form 3D structure of complex compound (sugars, enzymes, polysaccharides, lipids, nucleic acids, extracellular DNA structural proteins) which is used in energy exchange mechanism in response to environmental signals (Mishra, 2013) and have a direct role in heat stress tolerance (Nishihata et al., 2018; Ogden et al., 2019). These EPSs are involved in cell aggregation, adhesion, water retention, building a protective barrier and supplying nutrients. They contain high-molecular-weight polymers that enable bacteria to cling with the soil particles *via* hydrogen bonding, van der wall forces, anionic and cationic bonding, and keep them alive. The inoculation with heat-resilient, ESPs-producing *B. cereus* increased root and shoot length, chlorophyll and proline contents, water intake, number of flowers and fruits in tomato under high temperature (Mukhtar T. et al., 2020). HS induces changes in EPSs production as well as other cellular proteins, i.e., HSPs which prevents protein aggregation, misfolding, and target abnormally folded proteins for degradation (Parsell and Lindquist, 1993). Nguyen et al. (2014) reported that *Pseudomonas* sp. strain PsJN produced ESPs at high temperature and enhanced growth of the potato. Application of *Bacillus, Pseudomonas* and *Azospirillum* spp. (Ali et al., 2011; Abd El-Daim et al., 2019; Da Jeong Shin et al., 2019) have been shown to induce metabolic and regulatory proteins modulation to develop heat stress tolerance along with the cold and drought stresses. Apart from this, EPS act as chemoattractant and help to develop microbial biofilm matrix on the root surface that protect root surfaces from any damage under any kind of stress including HS.

#### Microbe-Induced HS Tolerance and Antioxidant Activity

Plant growth-promoting rhizobacteria not only produce phytohormones but also help plants by modulation of different genes under heat stress. Heat stress upregulates the genes involved in autophagy (e.g., *SlWRKY33b* and *SlATG5* genes) that are harmful to plants. But the application of *Bacillus cereus* sp. isolate (SA1) and humic acid (HA) down-regulate the expression of these genes which not only give relief but provide thermotolerance to the tomato plants (Khan et al., 2020) and increase the uptake of potassium (K) phosphorus (P), and iron (Fe). *B. cereus* SA1 enhanced the activity of antioxidants (CAT, APX, SOD) as well as the efficacy of chlorophyll pigments. The inoculation also resulted in a reduced level of abscisic acid (ABA) and enhanced the level of salicylic acid (SA). The treated plant showed a reduced level of glutathione (GSH). It has been shown that thermotolerant bacteria (*Bacillus* spp. *actinobacterium Kocuria* sp.) and cyanobacteria (*Calothrix elenkinii* and *Anabaena laxa*) show plant growth-promoting activities (30–50% root and shoot length, biomass, and dry weight) and stimulate peroxidase (5–10%) and endoglucanase activities in a range of different plants (Kumar et al., 2013).

Thermotolerant *P. putida* strain *AKMP7* inoculated to wheat plants increased the plant biomass and dry weight, enhanced root and shoot length, increase the number of tillers and spikelet, and enhance grain formation (Ali et al., 2011). The inoculated bacteria prevent the plant from cellular injury, enhance the antioxidant enzymatic activities (SOD, APX, CAT), and improve cellular metabolism, e.g., the level of protein, proline contents, cellular sugar, amino acid, and starch, and the efficiency of chlorophyll under heat stress condition. *AKMP7* formed biofilm on plant roots which help plants to combat adverse heat stress conditions.

## Conclusions and Future Prospects

Global warming has become a critical challenge to food security, causing severe yield losses of major crops worldwide. Given the expanding needs for global food supply as well as the extreme pressure of population growth and climate change trajectories, strategies should primarily be focused on the right investigation on different abiotic stresses especially on heat stress that becomes a key problem in the last decade. The plants are more vulnerable to high temperatures because of their sessile nature. They exhibit physio-morphological, biochemical, and molecular adaptations against heat, but further investigations are required to understand the thermotolerance mechanism active in different plant species. The plants themselves produce antioxidants, reduce the stomatal conductance, activate heat-responsive genes, and produce heat shock proteins, but on the other hand, exogenous or foliar application of chemicals like calcium chloride (CaCl_2_), salicylic acid, bio-stimulants, nanoparticles, and osmoprotectants are useful in sustainable agriculture. Furthermore, heat-sensitive genes can be targeted through CRISPR-Cas9 to develop heat-insensitive crops in the future. The heat resilient microbes produces phytohormones, ethylene, ACC deaminase, antioxidant enzymes, and HSP under heat stress enabling plants to maintain their growth under stress. A recent review publishes the meta-analysis of 39 published studies in support of PGPR-mediated thermotolerance in plants. This supports that microbe-mediated solutions are more sustainable than developing heat-tolerant varieties as microbes are usually present in the soil and rhizosphere and can form associations with homologous as well as non-homologous hosts. So, a single heat resilient microbe-inoculum may be effective in more than one crop or plants. Furthermore, the microbes are multi-purpose and exhibit many other traits of plant interest apart from giving tolerance against heat stress making the microbe-therapy more effective than chemical or genetic engineering or breeding approaches. However, the molecular mechanisms involved in this tolerance may be studied in detail in different crops prone to heat stress. As plants can't live in isolation in any environment, they have a direct impact of environment and also interact with the microbes present in soil. Whereas microbes also interact with other microbes and to the environment. So, this tri-partite association is very important for the stable functioning of the plants and microbes and over all agricultural sustainability in any given environment.

## Author Contributions

MA collected data, made illustrations and figures, and wrote the initial draft. MI, FM, and MS helped in the analysis and writing. AI conceptualized and finalized the review. All authors contributed to the article and approved the submitted version.

## Conflict of Interest

The authors declare that the research was conducted in the absence of any commercial or financial relationships that could be construed as a potential conflict of interest.

## Publisher's Note

All claims expressed in this article are solely those of the authors and do not necessarily represent those of their affiliated organizations, or those of the publisher, the editors and the reviewers. Any product that may be evaluated in this article, or claim that may be made by its manufacturer, is not guaranteed or endorsed by the publisher.

## References

[B1] Abd El-Daim I. Bejai S. Fridborg I. Meijer J. (2018). Identifying potential molecular factors involved in Bacillus amyloliquefaciens 5113 mediated abiotic stress tolerance in wheat. Plant Biol. 20, 271–279. 10.1111/plb.12680 29247572

[B2] Abd El-Daim I. A. Bejai S. Meijer J. (2014). Improved heat stress tolerance of wheat seedlings by bacterial seed treatment. Plant Soil 379, 337–350. 10.1007/s11104-014-2063-3

[B3] Abd El-Daim I. A. Bejai S. Meijer J. (2019). Bacillus velezensis 5113 induced metabolic and molecular reprogramming during abiotic stress tolerance in wheat. Sci. Rep. 9, 1–18. 10.1038/s41598-019-52567-x 31704956 PMC6841942

[B4] Ahamed K.-U. Nahar K. Fujita M. Hasanuzzaman M. (2010). Variation in plant growth, tiller dynamics and yield components of wheat (*Triticum aestivum* L.) due to high temperature stress. Adv. Agric. Botanics 2, 213–224.

[B5] Akter N. Islam M. R. (2017). Heat stress effects and management in wheat. A review. Agron. Sustain. Dev. 37, 37. 10.1007/s13593-017-0443-9

[B6] Alexandre A. Oliveira S. (2013). Response to temperature stress in rhizobia. Critic. Rev. Microbiol. 39, 219–228. 10.3109/1040841X.2012.702097 22823534

[B7] Ali S. Z. Sandhya V. Grover M. Linga V. R. Bandi V. (2011). Effect of inoculation with a thermotolerant plant growth promoting *Pseudomonas putida* strain AKMP7 on growth of wheat (*Triticum* spp.) under heat stress. J. Plant Interact. 6, 239–246. 10.1080/17429145.2010.545147

[B8] Al-Karaki G. N. (2012). Phenological development-yield relationships in durum wheat cultivars under late-season high-temperature stress in a semiarid environment. ISRN Agron. 2012, 456856. 10.5402/2012/456856

[B9] Álvarez-Pérez S. Lievens B. Fukami T. (2019). Yeast–bacterium interactions: the next frontier in nectar research. Trends Plant Sci. 24, 393–401. 10.1016/j.tplants.2019.01.012 30792076

[B10] Argosubekti N. (2020). “A review of heat stress signaling in plants,” in IOP Conference Series: Earth and Environmental Science. Bristol: IOP Publishing, 012041.

[B11] Ashraf M. Hafeez M. (2004). Thermotolerance of pearl millet and maize at early growth stages: growth and nutrient relations. Biol. Plant. 48, 81–86. 10.1023/B:BIOP.0000024279.44013.61

[B12] Asseng S. Ewert F. Martre P. Rötter R. P. Lobell D. B. Cammarano D.. (2015). Rising temperatures reduce global wheat production. Nat. Clim. Change 5, 143–147. 10.1038/nclimate2470

[B13] Bakker P. A. Pieterse C. M. de Jonge R. Berendsen R. L. (2018). The soil-borne legacy. Cell. 172, 1178–1180. 10.1016/j.cell.2018.02.024 29522740

[B14] Balla K. Bed,o Z. Veisz O. (2007). Heat stress induced changes in the activity of antioxidant enzymes in wheat. Cereal Res. Commun. 35, 197–200. 10.1556/CRC.35.2007.2.8

[B15] Balla K. Karsai I. Bónis P. Kiss T. Berki Z. Horváth Á.. (2019). Heat stress responses in a large set of winter wheat cultivars (*Triticum aestivum* L.) depend on the timing and duration of stress. PLoS One 14, e0222639. 10.1371/journal.pone.0222639 31539409 PMC6754161

[B16] Balla K. Rakszegi M. Li Z. Bekes F. Bencze S. Veisz O. (2011). Quality of winter wheat in relation to heat and drought shock after anthesis. Czech J. Food Sci. 29, 117–128. 10.17221/227/2010-CJFS

[B17] Baniwal S. Bharti K. Chan K. Fauth M. Ganguli A. Kotak S.. (2005). Heat stress response in plants: A complex game with chaperones and more than twenty heat stress transcription factors. J. Biosci. 29, 471–487. 10.1007/BF02712120 15625403

[B18] Barka E. A. Nowak J. Clément C. (2006). Enhancement of chilling resistance of inoculated grapevine plantlets with a plant growth-promoting rhizobacterium, Burkholderia phytofirmans strain PsJN. Appl. Environ. Microbiol. 72, 7246–7252. 10.1128/AEM.01047-06 16980419 PMC1636148

[B19] Bäurle I. (2016). Plant heat adaptation: priming in response to heat stress. F1000Research 5, F1000 Faculty Rev-694. 10.12688/f1000research.7526.1 27134736 PMC4837978

[B20] Begum N. Qin C. Ahanger M. A. Raza S. Khan M. I. Ashraf M.. (2019). Role of arbuscular mycorrhizal fungi in plant growth regulation: implications in abiotic stress tolerance. Front. Plant Sci. 10, 1068. 10.3389/fpls.2019.01068 31608075 PMC6761482

[B21] Bensalim S. Nowak J. Asiedu S. K. (1998). A plant growth promoting rhizobacterium and temperature effects on performance of 18 clones of potato. Am. J. Potato Res. 75, 145–152.

[B22] Binenbaum J. Weinstain R. Shani E. (2018). Gibberellin localization and transport in plants. Trends Plant Sci. 23, 410–421. 10.1016/j.tplants.2018.02.005 29530380

[B23] Bonde M. Nester S. Berner D. (2012). Effects of daily temperature highs on development of *Phakopsora pachyrhizi* on soybean. Phytopathology 102, 761–768. 10.1094/PHYTO-01-12-0011-R 22779743

[B24] Calvo P. Nelson L. Kloepper J. W. (2014). Agricultural uses of plant biostimulants. Plant Soil 383, 3–41. 10.1007/s11104-014-2131-8

[B25] Caverzan A. Casassola A. Brammer S. P. (2016). Antioxidant responses of wheat plants under stress. Genet. Mol. Biol. 39, 1–6. 10.1590/1678-4685-GMB-2015-0109 27007891 PMC4807390

[B26] Chakraborty D. Nagarajan S. Aggarwal P. Gupta V. Tomar R. Garg R.. (2008). Effect of mulching on soil and plant water status, and the growth and yield of wheat (*Triticum aestivum* L.) in a semi-arid environment. Agric. Water Manag. 95, 1323–1334. 10.1016/j.agwat.2008.06.001

[B27] Change I. C. (2014). Mitigation of Climate Change. Contribution of Working Group III to the Fifth Assessment Report of the Intergovernmental Panel on Climate Change. Geneva: IPCC, 1454.

[B28] Chen J. Zhou X. Wang W. Liu B. Lv Y. Yang W.. (2018). A review on fundamental of high entropy alloys with promising high–temperature properties. J. Alloys Compd. 760, 15–30. 10.1016/j.jallcom.2018.05.067

[B29] Chen Y. Liu T. Tian X. Wang X. Li M. Wang S.. (2015). Effects of plastic film combined with straw mulch on grain yield and water use efficiency of winter wheat in Loess Plateau. Field Crops Res. 172, 53–58. 10.1016/j.fcr.2014.11.016

[B30] Cheng L. Zou Y. Ding S. Zhang J. Yu X. Cao J.. (2009). Polyamine accumulation in transgenic tomato enhances the tolerance to high temperature stress. J. Integr. Plant Biol. 51, 489–499. 10.1111/j.1744-7909.2009.00816.x 19508360

[B31] Chialva M. Salvioli Di Fossalunga A. Daghino S. Ghignone S. Bagnaresi P. Chiapello M.. (2018). Native soils with their microbiotas elicit a state of alert in tomato plants. New Phytol. 220, 1296–1308. 10.1111/nph.15014 29424928

[B32] Commuri P. Jones R. (2001). High temperatures during endosperm cell division in maize: a genotypic comparison under in vitro and field conditions. Crop Sci. 41, 1122–1130. 10.2135/cropsci2001.4141122x

[B33] Cossani C. M. Reynolds M. P. (2012). Physiological traits for improving heat tolerance in wheat. Plant Physiol. 160, 1710–1718. 10.1104/pp.112.207753 23054564 PMC3510104

[B34] Da Costa M. V. J. Ramegowda Y. Ramegowda V. Karaba N. N. Sreeman S. M. Udayakumar M. (2021). Combined drought and heat stress in rice: responses, phenotyping and strategies to improve tolerance. Rice Sci. 28, 233–242. 10.1016/j.rsci.2021.04.003

[B35] Da Jeong Shin S.-J. Y. Hong J. K. Weon H.-Y. Song J. Sang M. K. (2019). Effect of Bacillus aryabhattai H26-2 and B. siamensis H30-3 on growth promotion and alleviation of heat and drought stresses in Chinese cabbage. Plant Pathol. J. 35, 178. 10.5423/PPJ.NT.08.2018.0159 31007648 PMC6464199

[B36] Damayanti T. Putra L. (2010). Hot water treatment of cutting-cane infected with Sugarcane streak mosaic virus (SCSMV). J. ISSAAS 16, 17–25. 10.5423/20113256683

[B37] Danish S. Zafar-Ul-Hye M. Mohsin F. Hussain M. (2020). ACC-deaminase producing plant growth promoting rhizobacteria and biochar mitigate adverse effects of drought stress on maize growth. PLoS ONE 15, e0230615. 10.1371/journal.pone.0230615 32251430 PMC7135286

[B38] Dastogeer K. M. Zahan M. I. Rhaman M. S. Sarker M. S. Chakraborty A. (2022). Microbe-mediated thermotolerance in plants and pertinent mechanisms-a meta-analysis and review. Front. Microbiol. 13, 833566. 10.3389/fmicb.2022.833566 35330772 PMC8940538

[B39] Devireddy A. R. Tschaplinski T. J. Tuskan G. A. Muchero W. Chen J.-G. (2021). Role of reactive oxygen species and hormones in plant responses to temperature changes. Int. J. Mol. Sci. 22, 8843. 10.3390/ijms22168843 34445546 PMC8396215

[B40] Dias A. Lidon F. (2009). Evaluation of grain filling rate and duration in bread and durum wheat, under heat stress after anthesis. J. Agron. Crop Sci. 195, 137–147. 10.1111/j.1439-037X.2008.00347.x

[B41] Dias A. S. Lidon F. C. (2010). Bread and durum wheat tolerance under heat stress: a synoptical overview. Emir. J. Food Agric. 2010, 412–436. 10.9755/ejfa.v22i6.4660

[B42] Dimitrov I. Trzebicka B. Müller A. H. Dworak A. Tsvetanov C. B. (2007). Thermosensitive water-soluble copolymers with doubly responsive reversibly interacting entities. Prog. Polym. Sci. 32, 1275–1343. 10.1016/j.progpolymsci.2007.07.001

[B43] Ding W. Song L. Wang X. Bi Y. (2010). Effect of abscisic acid on heat stress tolerance in the calli from two ecotypes of Phragmites communis. Biol. Plant. 54, 607–613. 10.1007/s10535-010-0110-3

[B44] Djanaguiraman M. Annie Sheeba J. Durga Devi D. Bangarusamy U. (2009). Cotton leaf senescence can be delayed by nitrophenolate spray through enhanced antioxidant defence system. J. Agron. Crop Sci. 195, 213–224. 10.1111/j.1439-037X.2009.00360.x

[B45] Djanaguiraman M. Narayanan S. Erdayani E. Prasad P. V. (2020). Effects of high temperature stress during anthesis and grain filling periods on photosynthesis, lipids and grain yield in wheat. BMC Plant Biol. 20, 1–12. 10.1186/s12870-020-02479-0 32517754 PMC7285450

[B46] Djanaguiraman M. Prasad P. Al-Khatib K. (2011). Ethylene perception inhibitor 1-MCP decreases oxidative damage of leaves through enhanced antioxidant defense mechanisms in soybean plants grown under high temperature stress. Environ. Exp. Bot. 71, 215–223. 10.1016/j.envexpbot.2010.12.006

[B47] Djanaguiraman M. Prasad P. V. Seppanen M. (2010). Selenium protects sorghum leaves from oxidative damage under high temperature stress by enhancing antioxidant defense system. Plant Physiol. Biochem. 48, 999–1007. 10.1016/j.plaphy.2010.09.009 20951054

[B48] Dodd I. C. Pérez-Alfocea F. (2012). Microbial amelioration of crop salinity stress. J. Exp. Bot. 63, 3415–3428. 10.1093/jxb/ers033 22403432

[B49] Dubey R. Pathak H. Chakrabarti B. Singh S. Gupta D. K. Harit R. (2020). Impact of terminal heat stress on wheat yield in India and options for adaptation. Agric. Syst. 181, 102826. 10.1016/j.agsy.2020.102826

[B50] Dupuis I. Dumas C. (1990). Influence of temperature stress on in vitro fertilization and heat shock protein synthesis in maize (*Zea mays* L.) reproductive tissues. Plant Physiol. 94, 665–670. 10.3389/fpls.2015.00999 16667763 PMC1077283

[B51] Durán P. Thiergart T. Garrido-Oter R. Agler M. Kemen E. Schulze-Lefert P.. (2018). Microbial interkingdom interactions in roots promote *Arabidopsis* survival. Cell 175, 973–983.e914. 10.1016/j.cell.2018.10.020 30388454 PMC6218654

[B52] Easterling W. E. Aggarwal P. K. Batima P. Brander K. M. Erda L. Howden S. M.. (2007). Food, fibre and forest products. Clim. Change 2007, 273–313.

[B53] Etesami H. Alikhani H. A. Hosseini H. M. (2015). Indole-3-acetic acid (IAA) production trait, a useful screening to select endophytic and rhizosphere competent bacteria for rice growth promoting agents. MethodsX. 2, 72–78. 10.1016/j.mex.2015.02.008 26150974 PMC4487705

[B54] Etesami H. Beattie G. A. (2017). “Plant-microbe interactions in adaptation of agricultural crops to abiotic stress conditions,” in Probiotics and Plant Health (Singapore: Springer), 163–200. 10.1007/978-981-10-3473-2_7

[B55] Etterson J. R. Shaw R. G. (2001). Constraint to adaptive evolution in response to global warming. Science 294, 151–154. 10.1126/science.1063656 11588260

[B56] Fahad S. Hussain S. Saud S. Hassan S. Ihsan Z. Shah A. N.. (2016). Exogenously applied plant growth regulators enhance the morpho-physiological growth and yield of rice under high temperature. Front. Plant Sci. 7, 1250. 10.3389/fpls.2016.01250 27625658 PMC5003834

[B57] Farooq M. Bramley H. Palta J. A. Siddique K. H. (2011). Heat stress in wheat during reproductive and grain-filling phases. Crit. Rev. Plant Sci. 30, 491–507. 10.1080/07352689.2011.615687

[B58] Finkelstein R. (2013). Abscisic acid synthesis and response. Am. Soci. Plant Biol. 11, 166. 10.1199/tab.0166 24273463 PMC3833200

[B59] Fitter A. H. Hay R. K. (2012). Environmental Physiology of Plants. New York: Academic press.

[B60] Friedlingstein P. Houghton R. Marland G. Hackler J. Boden T. A. Conway T.. (2010). Update on CO 2 emissions. Nat. Geosci. 3, 811–812. 10.1038/ngeo1022

[B61] Giri A. (2013). Effect of Acute Heat Stress on Nutrient Uptake by Plant Roots. Toledo: The University of Toledo.

[B62] Giri A. Heckathorn S. Mishra S. Krause C. (2017). Heat stress decreases levels of nutrient-uptake and-assimilation proteins in tomato roots. Plants 6, 6. 28106834 10.3390/plants6010006 PMC5371765

[B63] Głab T. Kulig B. (2008). Effect of mulch and tillage system on soil porosity under wheat (*Triticum aestivum*). Soil Tillage Res. 99, 169–178. 10.1016/j.still.2008.02.004

[B64] Grant R. Kimball B. Conley M. White J. Wall G. Ottman M. J. (2011). Controlled warming effects on wheat growth and yield: field measurements and modeling. Agron. J. 103, 1742–1754. 10.2134/agronj2011.0158

[B65] Gray S. B. Brady S. M. (2016). Plant developmental responses to climate change. Dev. Biol. 419, 64–77. 10.1016/j.ydbio.2016.07.023 27521050

[B66] Hakim M. Hossain A. Da Silva J. a.T Zvolinsky V. Khan M. (2012). Protein and starch content of 20 wheat (*Triticum aestivum* L.) genotypes exposed to high temperature under late sowing conditions. J. Sci. Res. 4, 477–477. 10.3329/jsr.v4i2.8679

[B67] Hakim S. Naqqash T. Nawaz M. S. Laraib I. Siddique M. J. Zia R.. (2021). Rhizosphere engineering with plant growth-promoting microorganisms for agriculture and ecological sustainability. Front. Sustain. Food Syst. 5, 16. 10.3389/fsufs.2021.617157

[B68] Hasanuzzaman M. Nahar K. Alam M. Roychowdhury R. Fujita M. (2013). Physiological, biochemical, and molecular mechanisms of heat stress tolerance in plants. Int. J. Mol. Sci. 14, 9643–9684. 10.3390/ijms14059643 23644891 PMC3676804

[B69] Hashem A. Abd Allah E. F. Alqarawi A. A. Al-Huqail A. A. Wirth S. Egamberdieva D. (2016). The interaction between arbuscular mycorrhizal fungi and endophytic bacteria enhances plant growth of Acacia gerrardii under salt stress. Front. Microbiol. 7, 1089. 10.3389/fmicb.2016.01089 27486442 PMC4949997

[B70] Hatfield J. L. Boote K. J. Kimball B. Ziska L. Izaurralde R. C. Ort D.. (2011). Climate impacts on agriculture: implications for crop production. Agron. J. 103, 351–370. 10.2134/agronj2010.0303

[B71] Hatfield J. L. Prueger J. H. (2015). Temperature extremes: effect on plant growth and development. Weather Clim. Extrem. 10, 4–10. 10.1016/j.wace.2015.08.001

[B72] Hemantaranjan A. Bhanu A. N. Singh M. Yadav D. Patel P. Singh R.. (2014). Heat stress responses and thermotolerance. Adv. Plants Agric. Res 1, 1–10. 10.15406/apar.2014.01.00012

[B73] Högy P. Poll C. Marhan S. Kandeler E. Fangmeier A. (2013). Impacts of temperature increase and change in precipitation pattern on crop yield and yield quality of barley. Food Chem. 136, 1470–1477. 10.1016/j.foodchem.2012.09.056 23194550

[B74] Hossain A. Da Silva J. T. (2012). Phenology, growth and yield of three wheat (*Triticum aestivum* L.) varieties as affected by high temperature stress. Not. Sci. Biol. 4, 97–109. 10.15835/nsb437879

[B75] Hovmøller M. S. Yahyaoui A. H. Milus E. A. Justesen A. F. (2008). Rapid global spread of two aggressive strains of a wheat rust fungus. Mol. Ecol. 17, 3818–3826. 10.1111/j.1365-294X.2008.03886.x 18673440

[B76] Howarth C. (2005). “Genetic improvements of tolerance to high temperature,” in Abiotic Stresses–Plant Resistance Through Breeding and Molecular Approaches, eds M. Ashraf, P. J. C. Harris (New York: The Haworth Press), 277–300.

[B77] Hsu S.-F. Lai H.-C. Jinn T.-L. (2010). Cytosol-localized heat shock factor-binding protein, AtHSBP, functions as a negative regulator of heat shock response by translocation to the nucleus and is required for seed development in Arabidopsis. Plant Physiol. 153, 773–784. 10.1104/pp.109.151225 20388662 PMC2879799

[B78] Hu S. Ding Y. Zhu C. (2020). Sensitivity and responses of chloroplasts to heat stress in plants. Front. Plant Sci. 11, 375. 10.3389/fpls.2020.00375 32300353 PMC7142257

[B79] Huang Y.-C. Niu C.-Y. Yang C.-R. Jinn T.-L. (2016). The heat stress factor HSFA6b connects ABA signaling and ABA-mediated heat responses. Plant Physiol. 172, 1182–1199. 10.1104/pp.16.00860 27493213 PMC5047099

[B80] Hurkman W. J. Tanaka C. K. Vensel W. H. Thilmony R. Altenbach S. B. (2013). Comparative proteomic analysis of the effect of temperature and fertilizer on gliadin and glutenin accumulation in the developing endosperm and flour from *Triticum aestivum* L. cv. Butte 86. Proteome Sci. 11, 1–15. 10.1186/1477-5956-11-8 23432757 PMC3599944

[B81] Hurkman W. J. Vensel W. H. Tanaka C. K. Whitehand L. Altenbach S. B. (2009). Effect of high temperature on albumin and globulin accumulation in the endosperm proteome of the developing wheat grain. J. Cereal Sci. 49, 12–23. 10.1016/j.jcs.2008.06.014

[B82] Hussain H. A. Men S. Hussain S. Chen Y. Ali S. Zhang S.. (2019). Interactive effects of drought and heat stresses on morpho-physiological attributes, yield, nutrient uptake and oxidative status in maize hybrids. Sci. Rep. 9, 1–12. 10.1038/s41598-019-40362-7 30846745 PMC6405865

[B83] Hussain S. Khaliq A. Mehmood U. Qadir T. Saqib M. Iqbal M. A.. (2018). Sugarcane Production Under Changing Climate: Effects of Environmental Vulnerabilities on Sugarcane Diseases, Insects and Weeds. Vienna: Intech Open.

[B84] Ikeda H. Kinoshita T. Yamamoto T. Yamasaki A. (2019). Sowing time and temperature influence bulb development in spring-sown onion (*Allium cepa* L.). Sci. Hortic. 244, 242–248. 10.1016/j.scienta.2018.09.050

[B85] Imran A. Hakim S. Tariq M Nawaz M. S. Laraib I. Gulzar U.. (2021). Diazotrophs for lowering nitrogen pollution crises; looking deep into the roots. Front. Microbiol. 12, 637815. 10.3389/fmicb.2021.637815 34108945 PMC8180554

[B86] Imran A. Mirza M. S. Shah T. M. Malik K. A. Hafeez F. Y. (2015). Differential response of kabuli and desi chickpea genotypes toward inoculation with PGPR in different soils. Front. Microbiol. 6, 859. 10.3389/fmicb.2015.00859 26379638 PMC4548240

[B87] Issa A. Esmaeel Q. Sanchez L. Courteaux B. Guise J.-F. Gibon Y.. (2018). Impacts of Paraburkholderia phytofirmans strain PsJN on tomato (*Lycopersicon esculentum* L.) under high temperature. Front. Plant Sci. 9, 1397. 10.3389/fpls.2018.01397 30405648 PMC6201190

[B88] Jegadeesan S. Chaturvedi P. Ghatak A. Pressman E. Meir S. Faigenboim A.. (2018). Proteomics of heat-stress and ethylene-mediated thermotolerance mechanisms in tomato pollen grains. Front. Plant Sci. 9, 1558. 10.3389/fpls.2018.01558 30483278 PMC6240657

[B89] Jones L. M. Koehler A. K. Trnka M. Balek J. Challinor A. J. Atkinson H. J.. (2017). Climate change is predicted to alter the current pest status of *Globodera pallida* and *G. rostochiensis* in the United Kingdom. Glob. Change Biol. 23, 4497–4507. 10.1111/gcb.13676 28261933 PMC5655772

[B90] Kachhap S. Chaudhary A. Singh S. (2015). Response of plant growth promoting rhizobacteria (pgpr) in relation to elevated temperature conditions in groundnut (*Arachis hypogaea* L.). Ecoscan 9, 771–778.

[B91] Kandil A. Attia A. Sharief A. Leilh A. (2011). Response of onion (*Allium cepa* L.) yield to water stress and mineral biofertilization. Acta. Agron. Hung. 59, 361–370. 10.1556/AAgr.59.2011.4.7

[B92] Kang S.-M. Khan A. L. Waqas M. You Y.-H. Hamayun M. Joo G.-J.. (2015). Gibberellin-producing Serratia nematodiphila PEJ1011 ameliorates low temperature stress in *Capsicum annuum* L. Eur. J. Soil Biol. 68, 85–93. 10.1016/j.ejsobi.2015.02.005

[B93] Kaushal N. Bhandari K. Siddique K. H. Nayyar H. (2016). Food crops face rising temperatures: an overview of responses, adaptive mechanisms, and approaches to improve heat tolerance. Cogent Food Agric. 2, 1134380. 10.1080/23311932.2015.1134380

[B94] Khan A. L. Hamayun M. Kim Y. H. Kang S. M. Lee J. H. Lee I. J. (2011). Gibberellins producing endophytic Aspergillus fumigatus sp. LH02 influenced endogenous phytohormonal levels, isoflavonoids production and plant growth in salinity stress. Process Biochem. 6, 440–447. 10.1016/j.procbio.2010.09.013

[B95] Khan M. A. Asaf S. Khan A. L. Jan R. Kang S.-M. Kim K.-M.. (2020). Extending thermotolerance to tomato seedlings by inoculation with SA1 isolate of Bacillus cereus and comparison with exogenous humic acid application. PLoS ONE 15, e0232228. 10.1371/journal.pone.0232228 32353077 PMC7192560

[B96] Khan M. I. R. Asgher M. Khan N. A. (2014). Alleviation of salt-induced photosynthesis and growth inhibition by salicylic acid involves glycinebetaine and ethylene in mungbean (*Vigna radiata* L.). Plant Physiol. Biochem. 80, 67–74. 10.1016/j.plaphy.2014.03.026 24727790

[B97] Khichar M. Niwas R. (2007). Thermal effect on growth and yield of wheat under different sowing environments and planting systems. Indian J. Agric. Sci. 41, 92–96.

[B98] Kmiecik P. Leonardelli M. Teige M. (2016). Novel connections in plant organellar signalling link different stress responses and signalling pathways. J. Exp. Bot. 67, 3793–3807. 10.1093/jxb/erw136 27053718

[B99] Kong B. Li Z. Wang E. Lu W. Chen L. Qi G. (2018). An experimental study for characterization the process of coal oxidation and spontaneous combustion by electromagnetic radiation technique. Process Saf. Environ. 119, 285–294. 10.1016/j.psep.2018.08.002

[B100] Kumar A. Verma J. P. (2018). Does plant-microbe interaction confer stress tolerance in plants: a review?. Microbiol. Res. 207, 41–52. 10.1016/j.micres.2017.11.004 29458867

[B101] Kumar M. Prasanna R. Bidyarani N. Babu S. Mishra B. K. Kumar A.. (2013). Evaluating the plant growth promoting ability of thermotolerant bacteria and cyanobacteria and their interactions with seed spice crops. Sci. Hortic. 164, 94–101. 10.1016/j.scienta.2013.09.014

[B102] Kumarathunge D. P. Medlyn B. E. Drake J. E. Tjoelker M. G. Aspinwall M. J. Battaglia M.. (2019). Acclimation and adaptation components of the temperature dependence of plant photosynthesis at the global scale. New phytol. 222, 768–784. 10.1111/nph.15668 30597597

[B103] Kwak M.-J. Kong H. G. Choi K. Kwon S.-K. Song J. Y. Lee J.. (2018). Rhizosphere microbiome structure alters to enable wilt resistance in tomato. Nat. Biotechnol. 36, 1100–1109. 10.1038/nbt.4232 30295674

[B104] Lamaoui M. Jemo M. Datla R. Bekkaoui F. (2018). Heat and drought stresses in crops and approaches for their mitigation. Front. Chem. 6, 26. 10.3389/fchem.2018.00026/full 29520357 PMC5827537

[B105] Lämke J. Bäurle I. (2017). Epigenetic and chromatin-based mechanisms in environmental stress adaptation and stress memory in plants. Genome Biol. 18, 1–11. 10.1186/s13059-017-1263-6 28655328 PMC5488299

[B106] Lang-Mladek C. Popova O. Kiok K. Berlinger M. Rakic B. Aufsatz W.. (2010). Transgenerational inheritance and resetting of stress-induced loss of epigenetic gene silencing in Arabidopsis. Mol. Plant. 3, 594–602. 10.1093/mp/ssq014 20410255 PMC2877484

[B107] LeBlanc C. Zhang F. Mendez J. Lozano Y. Chatpar K. Irish V. F.. (2018). Increased efficiency of targeted mutagenesis by CRISPR/Cas9 in plants using heat stress. Plant J. 93, 377–386. 29161464 10.1111/tpj.13782

[B108] Li N. Euring D. Cha J. Y. Lin Z. Lu M. Huang L.-J.. (2021). Plant hormone-mediated regulation of heat tolerance in response to global climate change. Front. Plant Sci. 11, 2318. 10.3389/fpls.2020.627969/full 33643337 PMC7905216

[B109] Li X. Cai C. Wang Z. Fan B. Zhu C. Chen Z. (2018). Plastid translation elongation factor Tu is prone to heat-induced aggregation despite its critical role in plant heat tolerance. Plant Physiol. 176, 3027–3045. 10.1104/pp.17.01672 29444814 PMC5884619

[B110] Li Z. Peng T. Xie Q. Han S. Tian J. (2010). Mapping of QTL for tiller number at different stages of growth in wheat using double haploid and immortalized F 2 populations. Genetic. 89, 409. 10.1007/s12041-010-0059-1 21273691

[B111] Li Z.-G. (2020). “Mechanisms of plant adaptation and tolerance to heat stress,” in Plant Ecophysiology and Adaptation Under Climate Change: Mechanisms and Perspectives II (Berlin: Springer), 39–59.

[B112] Liu C.-H. Siew W. Hung Y.-T. Jiang Y.-T. Huang C.-H. (2021). 1-Aminocyclopropane-1-carboxylate (ACC) deaminase gene in pseudomonas azotoformans is associated with the amelioration of salinity stress in tomato. J. Agric. Food Chem. 69, 913–921. 10.1021/acs.jafc.0c05628 33464897

[B113] Liu F. Xing S. Ma H. Du Z. Ma B. (2013). Cytokinin-producing, plant growth-promoting rhizobacteria that confer resistance to drought stress in *Platycladus orientalis* container seedlings. Appl. Microbiol. Biotechnol. 97, 9155–9164. 10.1007/s00253-013-5193-2 23982328

[B114] Liu H. Brettell L. E. (2019). Plant defense by VOC-induced microbial priming. Trends Plant Sci. 24, 187–189. 10.1016/j.tplants.2019.01.008 30738790

[B115] Liu Z. Tan V. Y. F. (2017). “Relative error bounds for nonnegative matrix factorization under a geometric assumption,” in 2017 IEEE International Conference on Acoustics, Speech and Signal Processing (ICASSP), 2552–2556.

[B116] Lobell D. B. Field C. B. (2007). Global scale climate–crop yield relationships and the impacts of recent warming. Environ. Res. Lett. 2, 014002. 10.1088/1748-9326/2/1/014002/meta15092357

[B117] Lobell D. B. Schlenker W. Costa-Roberts J. (2011). Climate trends and global crop production since 1980. Science 333, 616–620. 10.1126/science.1204531 21551030

[B118] Lu T. Ke M. Lavoie M. Jin Y. Fan X. Zhang Z.. (2018). Rhizosphere microorganisms can influence the timing of plant flowering. Microbiome 6, 1–12. 10.1186/s40168-018-0615-0 30587246 PMC6307273

[B119] Ma L. Qiao J. Kong X. Zou Y. Xu X. Chen X.. (2015). Effect of low temperature and wheat winter-hardiness on survival of Puccinia striiformis f. sp. tritici under controlled conditions. PLoS ONE 10, e0130691. 10.1371/journal.pone.0130691 26083371 PMC4470655

[B120] Maheshwari D. K. Dheeman S. Agarwal M. (2015). “VPhytohormone-producing PGPR for sustainable agriculture,” in Bacterial Metabolites in Sustainable Agroecosystem (Berlin: Springer), 159–182.

[B121] Majeed S. Rana I. A. Mubarik M. S. Atif R. M. Yang S.-H. Chung G.. (2021). Heat stress in cotton: a review on predicted and unpredicted growth-yield anomalies and mitigating breeding strategies. Agronomy 11, 1825. 10.3390/agronomy11091825

[B122] Mangrauthia S. K. Shakya V. P. S. Jain R. Praveen S. (2009). Ambient temperature perception in papaya for papaya ringspot virus interaction. Virus Genes 38, 429–434. 10.1007/s11262-009-0336-3 19247826

[B123] Marchand F. L. Mertens S. Kockelbergh F. Beyens L. Nijs I. (2005). Performance of High Arctic tundra plants improved during but deteriorated after exposure to a simulated extreme temperature event. Glob. Chang Biol. 11, 2078–2089. 10.1111/j.1365-2486.2005.01046.x 34991289

[B124] Mariette N. Androdias A. Mabon R. Corbiere R. Marquer B. Montarry J.. (2016). Local adaptation to temperature in populations and clonal lineages of the Irish potato famine pathogen Phytophthora infestans. Ecol. Evol. 6, 6320–6331. 10.1002/ece3.2282 27648246 PMC5016652

[B125] Marutani Y. Yamauchi Y. Kimura Y. Mizutani M. Sugimoto Y. (2012). Damage to photosystem II due to heat stress without light-driven electron flow: involvement of enhanced introduction of reducing power into thylakoid membranes. Planta 236, 753–761. 10.1007/s00425-012-1647-5 22526503

[B126] Mcclung C. R. Davis S. J. (2010). Ambient thermometers in plants: from physiological outputs towards mechanisms of thermal sensing. Curr. Biol. 20, R1086–R1092. 10.1016/j.cub.2010.10.035 21172632

[B127] Medina E. Kim S.-H. Yun M. Choi W.-G. (2021). Recapitulation of the function and role of ROS generated in response to heat stress in plants. Plants 10, 371. 10.3390/plants10020371 33671904 PMC7918971

[B128] Meinke H. Nelson R. Kokic P. Stone R. Selvaraju R. Baethgen W. (2006). Actionable climate knowledge: from analysis to synthesis. Clim. Res. 33, 101–110. 10.3354/cr033101

[B129] Mengutay M. Ceylan Y. Kutman U. B. Cakmak I. (2013). Adequate magnesium nutrition mitigates adverse effects of heat stress on maize and wheat. Plant soil 368, 57–72. 10.1007/s11104-013-1761-6

[B130] Miller P. Lanier W. Brandt S. (2001). Using Growing Degree Days to Predict Plant Stages. Bozeman: Montana State University-Bozeman, 994–2721.

[B131] Milus E. A. Kristensen K. Hovmøller M. S. (2009). Evidence for increased aggressiveness in a recent widespread strain of *Puccinia striiformis* f. sp. tritici causing stripe rust of wheat. Phytopathology 99, 89–94. 10.1094/PHYTO-99-1-0089 19055439

[B132] Mishra J. (2013). “Microbial exopolysaccharides,” in The Prokaryotes–Applied Bacteriology and Biotechnolgy, ED E. Rosenberg. Berlin: Springer-Verlag.

[B133] Mishra S. K. Khan M. H. Misra S. Dixit V. K. Khare P. Srivastava S.. (2017). Characterisation of Pseudomonas spp. and *Ochrobactrum* sp. isolated from volcanic soil. Antonie Van Leeuwenhoek 110, 253–270. 10.1007/s10482-016-0796-0 27853952

[B134] Mittler R. Vanderauwera S. Suzuki N. Miller G. Tognetti V. B. Vandepoele K.. (2011). ROS signaling: the new wave? Trends Plant Sci. 16, 300–309. 10.1016/j.tplants.2011.03.007 21482172

[B135] Modarresi M. Mohammadi V. Zali A. Mardi M. (2010). Response of wheat yield and yield related traits to high temperature. Cereal Res. Commun. 38, 23–31. 10.1556/CRC.38.2010.1.3

[B136] Mohammed A. R. Tarpley L. (2010). Effects of high night temperature and spikelet position on yield-related parameters of rice (*Oryza sativa* L.) plants. Eur. J. Agron. 33, 117–123. 10.1016/j.eja.2009.11.006

[B137] Momčilović I. (2019). Effects of heat stress on potato productivity and nutritive quality. Hrana i ishrana 60, 43–48. 10.5937/hraIsh1902043M

[B138] Morales D. Rodríguez P. Dell'amico J. Nicolas E. Torrecillas A. Sánchez-Blanco M. J. (2003). High-temperature preconditioning and thermal shock imposition affects water relations, gas exchange and root hydraulic conductivity in tomato. Biol. Plant. 47, 203. 10.1023/B:BIOP.0000022252.70836.fc

[B139] Mukhtar S. Zareen M. Khaliq Z. Mehnaz S. Malik K. (2020). Phylogenetic analysis of halophyte-associated rhizobacteria and effect of halotolerant and halophilic phosphate-solubilizing biofertilizers on maize growth under salinity stress conditions. J. Appl. Microbiol. 128, 556–573. 10.1111/jam.14497 31652362

[B140] Mukhtar T. Smith D. Sultan T. Seleiman M. F. Alsadon A. A. Ali S.. (2020). Mitigation of heat stress in *Solanum lycopersicum* L. by ACC-deaminase and exopolysaccharide producing Bacillus cereus: effects on biochemical profiling. Sustainability 12, 2159. 10.3390/su12062159

[B141] Nahar K. (2013). Castor bean (*Ricinus communis* L.)-a biofuel plant: morphological and physiological parameters propagated from seeds in Bangladesh. Asian Bus. Rev. 2, 64–66.

[B142] Nandy S. Pathak B. Zhao S. Srivastava V. (2019). Heat-shock-inducible CRISPR/Cas9 system generates heritable mutations in rice. Plant Dir. 3, e00145. 10.1002/pld3.145 31404128 PMC6603394

[B143] Nawaz M. S. Arshad A. Rajput L. Fatima K. Ullah S. Ahmad M.. (2020). Growth-stimulatory effect of quorum sensing signal molecule n-acyl-homoserine lactone-producing multi-trait *Aeromonas* spp. on wheat genotypes under salt stress. Front. Microbiol. 11, 553621. 10.3389/fmicb.2020.553621 33117303 PMC7550764

[B144] Nguyen H. T. Razafindralambo H. Blecker C. N'yapo C. Thonart P. Delvigne F. (2014). Stochastic exposure to sub-lethal high temperature enhances exopolysaccharides (EPS) excretion and improves Bifidobacterium bifidum cell survival to freeze–drying. Biochem. Eng. J. 88, 85–94. 10.1016/j.bej.2014.04.005

[B145] Nishihata S. Kondo T. Tanaka K. Ishikawa S. Takenaka S. Kang C.-M.. (2018). Bradyrhizobium diazoefficiens USDA110 PhaR functions for pleiotropic regulation of cellular processes besides PHB accumulation. BMC Microbiol. 18, 1–17. 10.1186/s12866-018-1317-2 30355296 PMC6201568

[B146] Ogata T. Ishizaki T. Fujita M. Fujita Y. (2020). CRISPR/Cas9-targeted mutagenesis of OsERA1 confers enhanced responses to abscisic acid and drought stress and increased primary root growth under nonstressed conditions in rice. PLoS ONE 15, e0243376. 10.1371/journal.pone.0243376 33270810 PMC7714338

[B147] Ogden A. J. Mcaleer J. M. Kahn M. L. (2019). Characterization of the Sinorhizobium meliloti HslUV and ClpXP protease systems in free-living and symbiotic states. J. Bacteriol. 201, e00498–e00418. 10.1128/JB.00498-18 30670545 PMC6416911

[B148] Oleńska E. Małek W. Wójcik M. Swiecicka I. Thijs S. Vangronsveld J. (2020). Beneficial features of plant growth-promoting rhizobacteria for improving plant growth and health in challenging conditions: A methodical review. Sci. Total Environ. 2020, 140682. 10.1016/j.scitotenv.2020.140682 32758827

[B149] Ortiz R. Sayre K. D. Govaerts B. Gupta R. Subbarao G. Ban T.. (2008). Climate change: can wheat beat the heat? Agric. Ecosyst. Environ. 126, 46–58. 10.1016/j.agee.2008.01.01933594449

[B150] Osugi A. Sakakibara H. (2015). QandA: How do plants respond to cytokinins and what is their importance? BMC Biol. 13, 1–10. 10.1186/s12915-015-0214-5 26614311 PMC4662812

[B151] Park H. Weier S. Razvi F. Peña P. A. Sims N. A. Lowell J.. (2017). Towards the development of a sustainable soya bean-based feedstock for aquaculture. Plant Biotechnol. J. 15, 227–236. 10.1111/pbi.12608 27496594 PMC5258864

[B152] Parsell D. Lindquist S. (1993). The function of heat-shock proteins in stress tolerance: degradation and reactivation of damaged proteins. Annu. Rev. Genet. 27, 437–496. 8122909 10.1146/annurev.ge.27.120193.002253

[B153] Porter J. R. Xie L. Challinor A. J. Cochrane K. Howden S. M. Iqbal M. M.. (2014). Food Security and Food Production Systems. IPPC. Available online at: https://www.ipcc.ch/site/assets/uploads/2018/02/WGIIAR5-Chap7_FINAL.pdf (accessed May 9, 2022).

[B154] Prasad P. Boote K. Allen Jr L. Sheehy J. Thomas J. (2006). Species, ecotype and cultivar differences in spikelet fertility and harvest index of rice in response to high temperature stress. Field Crops Res. 95, 398–411. 10.1016/j.fcr.2005.04.008Get

[B155] Prasad P. Staggenborg S. Ristic Z. (2008). Impacts of Drought and/or Heat Stress on Physiological, Developmental, Growth, and Yield Processes of Crop Plants. Response of Crops to Limited Water: Understanding and Modeling Water Stress Effects on Plant Growth Processes 1, 301–355.

[B156] Prerostova S. Dobrev P. I. Kramna B. Gaudinova A. Knirsch V. Spichal L.. (2020). Heat acclimation and inhibition of cytokinin degradation positively affect heat stress tolerance of Arabidopsis. Front. Plant Sci 11, 87. 10.3389/fpls.2020.00087 32133021 PMC7040172

[B157] Rahman M. A. Chikushi J. Yoshida S. Karim A. (2009). Growth and yield components of wheat genotypes exposed to high temperature stress under control environment. Bangl. J. Agr. Res. 34, 360–372. 10.3329/bjar.v34i3.3961

[B158] Ramadoss D. Lakkineni V. K. Bose P. Ali S. Annapurna K. (2013). Mitigation of salt stress in wheat seedlings by halotolerant bacteria isolated from saline habitats. SpringerPlus 2, 6. 10.1186/2193-1801-2-6 23449812 PMC3579424

[B159] Rangan P. Subramani R. Kumar R. Singh A. K. Singh R. (2014). Recent advances in polyamine metabolism and abiotic stress tolerance. Biomed. Res. Int. 2014, 239621. 10.1155/2014/239621 25136565 PMC4124767

[B160] Rasmusson L. M. Buapet P. George R. Gullström M. Gunnarsson P. C. Björk M. (2020). Effects of temperature and hypoxia on respiration, photorespiration, and photosynthesis of seagrass leaves from contrasting temperature regimes. ICES J. Marine Sci. 77, 2056–2065. 10.1093/icesjms/fsaa093

[B161] Ratnarajah V. Gnanachelvam N. (2021). Effect of abiotic stress on onion yield: a review. Adv. Technol. 2021, 147–160.

[B162] Reddy P. S. Chakradhar T. Reddy R. A. Nitnavare R. B. Mahanty S. Reddy M. K. (2016). “Role of heat shock proteins in improving heat stress tolerance in crop plants,” in Heat Shock Proteins and Plants. (Berlin: Springer), 283–307.

[B163] Reynolds M. Calderini D. Condon A. Rajaram S. (2001). “Physiological basis of yield gains in wheat associated with the LR19 translocation from *Agropyron elongatum*,” in Wheat in a Global Environment (Berlin: Springer), 345–351.

[B164] Ritchie F. Bain R. Mcquilken M. (2013). Survival of sclerotia of R hizoctonia solani AG 3 PT and effect of soil-borne inoculum density on disease development on potato. J. Phytopathol. 161, 180–189. 10.1111/jph.12052

[B165] Roberts K. E. Hadfield J. D. Sharma M. D. Longdon B. (2018). Changes in temperature alter the potential outcomes of virus host shifts. PLoS Pathog. 14, e1007185. 30339695 10.1371/journal.ppat.1007185 PMC6209381

[B166] Ruelland E. Zachowski A. (2010). How plants sense temperature. Environ. Exp. Bot. 69, 225–232. 10.1016/j.envexpbot.2010.05.011

[B167] Rykaczewska K. (2015). The effect of high temperature occurring in subsequent stages of plant development on potato yield and tuber physiological defects. Am. J. Potato Res. 92, 339–349. 10.1007/s12230-015-9436-x

[B168] Sadowsky M. J. (2005). “Soil stress factors influencing symbiotic nitrogen fixation,” in Nitrogen Fixation in Agriculture, Forestry, Ecology, and the Environment (Berlin: Springer), 89–112.

[B169] Saleem M. Arshad M. Hussain S. Bhatti A. S. (2007). Perspective of plant growth promoting rhizobacteria (PGPR) containing ACC deaminase in stress agriculture. J. Ind. Microbiol. Biot. 34, 635–648. 10.1104/pp.126.3.1281 17665234

[B170] Sani E. Herzyk P. Perrella G. Colot V. Amtmann A. (2013). Hyperosmotic priming of Arabidopsis seedlings establishes a long-term somatic memory accompanied by specific changes of the epigenome. Genome Biol. 14, 1–24. 10.1186/gb-2013-14-6-r59 23767915 PMC3707022

[B171] Santos-Medellín C. Edwards J. Liechty Z. Nguyen B. Sundaresan V. (2017). Drought stress results in a compartment-specific restructuring of the rice root-associated microbiomes. MBio 8, e764. 10.1128/mBio.00764-17 28720730 PMC5516253

[B172] Sarieva G. Kenzhebaeva S. Lichtenthaler H. (2010). Adaptation potential of photosynthesis in wheat cultivars with a capability of leaf rolling under high temperature conditions. Russ. J. Plant Physl. 57, 28–36. 10.1134/S1021443710010048

[B173] Sarkar N. K. Kundnani P. Grover A. (2013). Functional analysis of Hsp70 superfamily proteins of rice (*Oryza sativa*). Cell Stress Chaperon 18, 427–437. 10.1007/s12192-012-0395-6 23264228 PMC3682022

[B174] Sarsu F. (2018). “Screening protocols for heat tolerance in rice at the seedling and reproductive stages,” in Pre-Field Screening Protocols for Heat-Tolerant Mutants in Rice (Berlin: Springer), 9–24.

[B175] Savada R. P. Ozga J. A. Jayasinghege C. P. Waduthanthri K. D. Reinecke D. M. (2017). Heat stress differentially modifies ethylene biosynthesis and signaling in pea floral and fruit tissues. Plant Mol. Biol. 95, 313–331. 10.1007/s11103-017-0653-1 28861701

[B176] Savicka M. Škute N. (2010). Effects of high temperature on malondialdehyde content, superoxide production and growth changes in wheat seedlings (*Triticum aestivum* L.). Ekologija 56, 26–33. 10.2478/v10055-010-0004-x

[B177] Sharma N. Chahal K. (2012). Protectant potential of Marigold oil (*Tagetes erecta* L.) and its fractions against *Tribolium castaneum* (Herbst). Ann. Plant. Protect Sci. 20, 294–297.

[B178] Shekhawat K. Saad M. M. Sheikh A. Mariappan K. Al-Mahmoudi H. Abdulhakim F.. (2021). Root endophyte induced plant thermotolerance by constitutive chromatin modification at heat stress memory gene loci. EMBO Rep. 22, e51049. 10.15252/embr.202051049 33426785 PMC7926228

[B179] Shrivastava P. Kumar R. (2015). Soil salinity: a serious environmental issue and plant growth promoting bacteria as one of the tools for its alleviation. Saudi J. Biol. Sci. 22, 123–131. 10.1016/j.sjbs.2014.12.001 25737642 PMC4336437

[B180] Siddiqui M. H. Al-Khaishany M. Y. Al-Qutami M. A. Al-Whaibi M. H. Grover A. Ali H. M.. (2015). Morphological and physiological characterization of different genotypes of faba bean under heat stress. Saudi J. Biol. Sci. 22, 656–663. 10.1016/j.sjbs.2015.06.002 26288573 PMC4537876

[B181] Singh B. Pathak K. Verma A. Verma V. Deka B. (2011). Effects of vermicompost, fertilizer and mulch on plant growth, nodulation and pod yield of French bean (*Phaseolus vulgaris* L.). Veg. Crop. Res. Bull. 74, 153–165. 10.2478/v10032-011-0013-7

[B182] Singh R. P. Jha P. Jha P. N. (2015). The plant-growth-promoting bacterium Klebsiella sp. SBP-8 confers induced systemic tolerance in wheat (*Triticum aestivum*) under salt stress. J. Plant Physiol. 184, 57–67. 10.1016/j.jplph.2015.07.002 26217911

[B183] Sita K. Sehgal A. Hanumantharao B. Nair R. M. Vara Prasad P. V. Kumar S.. (2017). Food Legumes and Rising Temperatures: Effects, Adaptive Functional Mechanisms Specific to Reproductive Growth Stage and Strategies to Improve Heat Tolerance. Front. Plant Sci. 8, 1658. 10.3389/fpls.2017.01658 29123532 PMC5662899

[B184] Sorty A. M. Meena K. K. Choudhary K. Bitla U. M. Minhas P. S. Krishnani K. K. (2016). Effect of plant growth promoting bacteria associated with halophytic weed (Psoralea corylifolia L) on germination and seedling growth of wheat under saline conditions. Appl. Biochem. Biotechnol. 180, 872–882. 10.1007/s12010-016-2139-z 27215915

[B185] Spaepen S. Dobbelaere S. Croonenborghs A. Vanderleyden J. (2008). Effects of Azospirillum brasilense indole-3-acetic acid production on inoculated wheat plants. Plant Soil. 312, 15–23. 10.1007/s11104-008-9560-1

[B186] Sun A.-Z. Guo F.-Q. (2016). Chloroplast retrograde regulation of heat stress responses in plants. Front. Plant Sci. 7, 398. doi|: 10.3389/fpls.2016.00398/full 27066042 PMC4814484

[B187] Sutherland I. W. (2001). Microbial polysaccharides from Gram-negative bacteria. Int. Dairy J. 11, 663–674. 10.1016/S0958-6946(01)00112-1

[B188] Suwa R. Hakata H. Hara H. El-Shemy H. A. Adu-Gyamfi J. J. Nguyen N. T.. (2010). High temperature effects on photosynthate partitioning and sugar metabolism during ear expansion in maize (*Zea mays* L.) genotypes. Plant Physiol. Bioch. 48, 124–130. 10.1016/j.plaphy.2009.12.010 20106675

[B189] Suzuki N. Koussevitzky S. Mittler R. Miller G. (2012). ROS and redox signalling in the response of plants to abiotic stress. Plant Cell Environ. 35, 259–270. 10.1111/j.1365-3040.2011.02336.x 21486305

[B190] Tian F. Li B. Ji B. Zhang G. Luo Y. (2009). Identification and structure–activity relationship of gallotannins separated from Galla chinensis. LWT-Food Sci. Technol. 42, 1289–1295. 10.1016/j.lwt.2009.03.004

[B191] Timm C. M. Carter K. R. Carrell A. A. Jun S.-R. Jawdy S. S. Vélez J. M.. (2018). Abiotic stresses shift belowground Populus-associated bacteria toward a core stress microbiome. MSystems 3. 10.1128/mSystems.00070-17 29404422 PMC5781258

[B192] Toh S. Imamura A. Watanabe A. Nakabayashi K. Okamoto M. Jikumaru Y.. (2008). High temperature-induced abscisic acid biosynthesis and its role in the inhibition of gibberellin action in Arabidopsis seeds. Plant Physiol. 146, 1368–1385. 10.1104/pp.107.113738 18162586 PMC2259091

[B193] Tubiello F. N. Soussana J.-F. Howden S. M. (2007). Crop and pasture response to climate change. Proc. Natl. Acad. Sci. 104, 19686–19690. 10.1073/pnas.0701728104 18077401 PMC2148358

[B194] Turkensteen L. Flier W. Wanningen R. Mulder A. (2000). Production, survival and infectivity of oospores of Phytophthora infestans. Plant Physiol. 49, 688–696. 10.1046/j.1365-3059.2000.00515.x

[B195] Uprety D. C. Reddy V. (2016). “Mitigation technologies to control high-temperature stress in crop plants,” in Crop Responses to Global Warming. (Berlin: Springer), 117–125.

[B196] van Es S. W. (2020). Too hot to handle, the adverse effect of heat stress on crop yield. Physiol. Plant. 169, 499–500. 10.1111/ppl.13165 32729121

[B197] Van Oosten M. J. Pepe O. De Pascale S. Silletti S. Maggio A. (2017). The role of biostimulants and bioeffectors as alleviators of abiotic stress in crop plants. Chem. Biol. Technol. 4, 5. 10.1186/s40538-017-0089-5

[B198] Vasseur F. Pantin F. Vile D. (2011). Changes in light intensity reveal a major role for carbon balance in Arabidopsis responses to high temperature. Plant Cell Environ. 34, 1563–1576. 10.1111/j.1365-3040.2011.02353.x 21707647

[B199] Velásquez A. C. Castroverde C. D. M. He S. Y. (2018). Plant–pathogen warfare under changing climate conditions. Curr Biol. 28, R619–R634. 10.1016/j.cub.2018.03.054 29787730 PMC5967643

[B200] Vollenweider P. Günthardt-Goerg M. S. (2005). Diagnosis of abiotic and biotic stress factors using the visible symptoms in foliage. Environ. Pollut. 137, 455–465. 10.1016/j.envpol.2005.01.032 16005758

[B201] Wahid A. Close T. (2007). Expression of dehydrins under heat stress and their relationship with water relations of sugarcane leaves. Biol. Plant. 51, 104–109. 10.1007/s10535-007-0021-0

[B202] Wahid A. Gelani S. Ashraf M. Foolad M. R. (2007). Heat tolerance in plants: an overview. Environ. Exp. Bot. 61, 199–223. 10.1016/j.envexpbot.2007.05.011

[B203] Wani S. H. Kumar V. Shriram V. Sah S. K. (2016). Phytohormones and their metabolic engineering for abiotic stress tolerance in crop plants. Crop J. 4, 162–176. 10.1016/j.cj.2016.01.010

[B204] Waqas M. Khan A. L. Kamran M. Hamayun M. Kang S.-M. Kim Y.-H.. (2012). Endophytic fungi produce gibberellins and indoleacetic acid and promotes host-plant growth during stress. Molecules 17, 10754–10773. 10.3390/molecules170910754 22960869 PMC6268353

[B205] Waraich E. Ahmad R. Halim A. Aziz T. (2012). Alleviation of temperature stress by nutrient management in crop plants: a review. J. Soil Sci. Plant Nut. 12, 221–244. 10.4067/S0718-95162012000200003

[B206] Wu C. Cui K. Wang W. Li Q. Fahad S. Hu Q.. (2016). Heat-induced phytohormone changes are associated with disrupted early reproductive development and reduced yield in rice. Sci. Rep. 6, 1–14. 10.1038/srep34978 27713528 PMC5054528

[B207] Wu H.-C. Bulgakov V. P. Jinn T.-L. (2018). Pectin methylesterases: cell wall remodeling proteins are required for plant response to heat stress. Front. Plant Sci. 9, 1612. 10.3389/fpls.2018.01612 30459794 PMC6232315

[B208] Xu Y. Chu C. Yao S. (2021). The impact of high-temperature stress on rice: challenges and solutions. Crop J. 9, 963–976. 10.1016/j.cj.2021.02.011

[B209] Xu Z. Liu S. Liu H. Yang C. Kang Y. Wang M. (2015). Unimolecular micelles of amphiphilic cyclodextrin-core star-like block copolymers for anticancer drug delivery. Chem. Commun. 51, 15768–15771. 10.1039/C5CC02743H 26121632

[B210] Yamauchi Y. (2018). “Integrated chemical control of abiotic stress tolerance using biostimulants,” in Plant, Abiotic Stress and Responses to Climate Change, 133–143.

[B211] Yang J. Kloepper J. W. Ryu C.-M. (2009). Rhizosphere bacteria help plants tolerate abiotic stress. Trends Plant Sci. 14, 1–4. 10.1016/j.tplants.2008.10.004 19056309

[B212] Yeh C.-H. Kaplinsky N. J. Hu C. Charng Y.-Y. (2012). Some like it hot, some like it warm: phenotyping to explore thermotolerance diversity. Plant Sci. 195, 10–23. 10.1111/nph.14212 22920995 PMC3430125

[B213] Yin Y. Li S. Liao W. Lu Q. Wen X. Lu C. (2010). Photosystem II photochemistry, photoinhibition, and the xanthophyll cycle in heat-stressed rice leaves. J. Plant Physiol. 167, 959–966. 10.1016/j.jplph.2009.12.021 20417985

[B214] Zafar S. A. Noor M. A. Waqas M. A. Wang X. Shaheen T. Raza M.. (2018). “Temperature extremes in cotton production and mitigation strategies,” in Past, Present and Future Trends in Cotton Breeding. eds M. R. Rahman and Y. Zafar (New York: Intechopen), 65-91.

[B215] Zahran H. Räsänen L. A. Karsisto M. Lindström K. (1994). Alteration of lipopolysaccharide and protein profiles in SDS-PAGE of rhizobia by osmotic and heat stress. World J. Microbiol. Biotechnol. 10, 100–105. 10.1007/BF00357572 24420895

[B216] Zhang X. Cai J. Wollenweber B. Liu F. Dai T. Cao W.. (2013). Multiple heat and drought events affect grain yield and accumulations of high molecular weight glutenin subunits and glutenin macropolymers in wheat. J. Cereal Sci. 57, 134–140. 10.1016/j.jcs.2012.10.010

[B217] Zhao D. Li Y.-R. (2015). Climate change and sugarcane production: potential impact and mitigation strategies. Int. J. Agron. 2015, 547386. 10.1155/2015/547386

[B218] Zhongming Z. Wei L. (2021). How to Beat the Heat: Memory Mechanism Allows Plants to Adapt to Heat Stress. Science Daily.

[B219] Zia R. Nawaz M. Yousaf S. Amin I. Hakim S. Mirza M.. (2021). Seed inoculation of desert-PGPR induce biochemical alterations and develop resistance against water stress in wheat. Physiol. Plant. 172:990–1006. 10.1111/ppl.13362 33547812

[B220] Zinn K. E. Tunc-Ozdemir M. Harper J. F. (2010). Temperature stress and plant sexual reproduction: uncovering the weakest links. J. Exp. Bot. 61, 1959–1968. 10.1093/jxb/erq053 20351019 PMC2917059

